# Written Languaging with Direct Feedback in Beginner CSL Writing: A Crossover Study of Metacognitive Engagement and Transfer Effects

**DOI:** 10.3390/jintelligence14090225

**Published:** 2026-09-19

**Authors:** Ming Lyu, Baoqian Yang, Wenting He

**Affiliations:** 1 School of International Chinese Language Education, Northeast Normal University, Changchun 130117, China; 2 Faculty of Education, Northeast Normal University, Changchun 130024, China

**Keywords:** cognitive architecture, metacognition, written languaging, individual differences, AI-assisted language learning, Chinese as a second language

## Abstract

Understanding the cognitive architectures that enable learners to process feedback and self-regulate is fundamental to fostering intelligent second language (L2) learning, particularly as AI-based pedagogies become prevalent. Direct written corrective feedback, while common in beginner L2 writing, often triggers only surface-level processing. This study investigates whether written languaging (WL), a metacognitive activity where learners explain language problems in writing, can deepen feedback processing, thereby activating a more robust cognitive architecture for error correction. Using a within-subjects crossover design with 15 beginner Chinese-as-a-second-language (CSL) learners from a UK secondary school, we compared the immediate and transfer effects of direct feedback with WL versus direct feedback only. Results showed that the WL condition significantly reduced errors per 100 characters (Z = −2.556, *p* = .011, r = 0.66), with 86.7% of participants showing improvement, indicating enhanced cognitive regulation at the local level. However, the effect was hierarchical, with no significant impact on clause-level accuracy. General Certificate of Secondary Education (GCSE) writing scores improved significantly from pre-to-post-test (Z = −3.342, *p* = .001, r = 0.89), demonstrating transfer to subsequent performance. Qualitative analyses revealed that learners’ attention focused predominantly on characters (48.4%) and vocabulary (29.8%), with engagement moderated by proficiency and motivation. This study provides empirical evidence for a cognitive architecture of feedback processing, wherein WL functions as a metacognitive amplifier. This architecture is operationalised as a three-stage processing sequence noticing, hypothesis-testing, and metalinguistic reflection, with writing accuracy and WL texts serving as observable proxies for the underlying cognitive processes. Specifically, our findings reveal that WL’s facilitative effects are hierarchical, strongest at the level of local error reduction and not yet extending to clause-level syntactic accuracy, and are moderated by individual differences in proficiency and motivation. These empirical insights offer a cognitive-psychological foundation that could inform the future design of adaptive feedback systems; however, as this study did not involve any AI system, these implications are theoretical and await empirical validation in AI-mediated learning environments.

## 1. Introduction

The rapid proliferation of artificial intelligence (AI) in education is fundamentally reshaping the landscape of second language (L2) learning. Intelligent tutoring systems, automated writing evaluation (AWE) tools, and generative AI platforms such as ChatGPT now offer unprecedented opportunities for personalized, adaptive, and scalable instruction (Kohnke et al., 2023). These tools can provide instantaneous, individualized feedback to L2 writers, raising important questions about how such technologies can be optimally harnessed to support language development. However, the effectiveness of these technological interventions ultimately hinges on a deeper, more fundamental question: what cognitive architectures enable human learners to perceive, process, and internalize feedback effectively? Understanding these underlying cognitive mechanisms is a prerequisite for designing AI systems that do not merely deliver information but genuinely catalyze language development. As Godwin-Jones (2022) observed, the integration of intelligent writing assistance into L2 learning requires not only technological sophistication but also a robust theoretical understanding of how learners cognitively engage with feedback a gap that the present study seeks to address by empirically investigating the metacognitive processes that underpin successful feedback processing. To move beyond the prevailing technology-driven paradigm, we must first reverse-engineer the cognitive architecture that learners naturally engage when processing feedback effectively. Rather than testing an AI tool, the present study empirically investigates how beginner Chinese-as-a-second-language (CSL) writers process feedback through written languaging (WL), thereby identifying the cognitive-psychological mechanisms that could inform the design of intelligent, adaptive AI systems. Specifically, our findings highlight the learner’s capacity for noticing, hypothesis-testing, and metalinguistic reflection as key processes that such systems might aim to scaffold. These implications, however, remain theoretical and are not directly tested in this human-feedback study. This foundational inquiry is a prerequisite for designing systems that genuinely catalyze language development, rather than merely delivering corrected output. The design of current AI-powered writing assistants, while technologically sophisticated, has largely been technology-driven rather than theory-driven. Most AWE systems and generative AI feedback tools operate on a “detect-and-correct” paradigm: they excel at identifying errors and providing correct forms, but treat feedback as a product to be delivered rather than a process to be cognitively processed (H. Chen & Pan, 2022; H. Yang et al., 2024). This implicit assumption overlooks a fundamental cognitive reality: durable learning does not arise from the correction itself, but from the learner’s metacognitive engagement with that correction (Ferris, 2004; Swain, 2006). By empirically investigating how beginner CSL writers process feedback through WL, this study does not test an AI tool, but rather provides the cognitive-psychological “functional specification” for what an intelligent, adaptive AI system must scaffold: the learner’s capacity for noticing, hypothesis-testing, and metalinguistic reflection.

L2 writing represents a particularly rich context for investigating this question. It is a cognitively demanding task that requires the intricate coordination of multiple dimensions of intelligence, including linguistic aptitude, working memory, metacognitive regulation, motivation, and emotional control (Skehan, 2015; Oxford, 2017). Within this complex landscape, written corrective feedback (WCF) has remained one of the most extensively studied areas in applied linguistics. Direct WCF where errors are explicitly identified and correct forms are provided is a staple in beginner-level L2 writing classrooms due to its clarity and efficiency (Ferris, 2010; Ellis, 2009). For lower-proficiency learners, direct feedback reduces the cognitive load during revision, allowing them to make corrections without having to infer the correct form themselves (Ferris, 2002). Meta-analyses have confirmed that WCF can significantly improve L2 written accuracy, with effects moderated by feedback type, learner proficiency, and instructional conditions. Brown et al. (2023), in a Bayesian meta-analysis, further underscored that the effectiveness of WCF is contingent upon how it is delivered and processed.

Yet a persistent pedagogical paradox remains: learners often correct errors locally based on direct feedback but fail to internalize the corrections, leading to recurrent errors in subsequent writing (Truscott, 1996; Ferris, 2004). This suggests that the cognitive architecture required for durable learning is not automatically activated by feedback provision alone. A crucial processing gap exists between “being told the correct form“ and “restructuring internal linguistic knowledge.” Ferris (2004) argued that the long-term effect of feedback depends not on what the teacher “provides,” but rather on how the learner “processes the received feedback.” However, what constitutes “effective processing” has not been fully defined at the operational level particularly in a logographic writing system like Chinese, where the cognitive load imposed by orthographic processing is fundamentally different from that in alphabetic L2 contexts (J. Yang, 2018, 2022). Truscott’s (1996) seminal critique of grammar correction, while controversial, highlighted a valid concern: without deeper cognitive engagement, error correction may remain superficial and fail to promote durable acquisition.

It is precisely on this cognitive gap that Swain’s (2006) concept of “languaging” offers a crucial theoretical pathway. Languaging the process of using language to mediate and reflect on one’s own cognitive activities is hypothesized to be a core component of an intelligent learner’s cognitive architecture. Swain (2006, p. 98) defined languaging as “the process of making meaning and shaping knowledge and experience through language”. When learners articulate their reasoning about why an error occurred and how to correct it, they engage in deeper cognitive processing, activating the noticing, hypothesis-testing, and metalinguistic functions essential for acquisition (Swain & Lapkin, 1995; Qi & Lapkin, 2001). Within Swain and Lapkin’s (1995) Output Hypothesis framework, output serves three key functions, noticing, hypothesis-testing, and metalinguistic reflection, which collectively transform feedback reception into active cognitive processing. WL, in particular, provides learners with extended processing time and a tangible written record for later retrieval, making it an ideal medium for studying this metacognitive dimension of feedback processing (Ishikawa, 2013; Suzuki, 2012). Recent research has further illuminated the mediating roles of individual difference factors such as language aptitude in the effectiveness of WL. Ishikawa and Suzuki (2023) found that the benefits of WL are not uniform across learners but are moderated by their cognitive aptitudes, underscoring the need to examine WL within the broader framework of individual differences in cognitive architecture.

Despite its theoretical appeal, the empirical exploration of WL has been largely confined to adult learners of Indo-European languages in English-as-a-Foreign-Language (EFL) contexts. A scoping review of languaging research in L2 education (C. Li et al., 2023) confirms that the majority of empirical studies have focused on university-level learners and alphabetic target languages. Two significant gaps exist in the literature, which this study addresses.

First, target language typology. Chinese, with its logographic writing system, imposes unique cognitive demands on orthographic processing. Unlike alphabetic scripts where errors predominantly occur at the morphosyntactic level (e.g., verb tense, subject-verb agreement), Chinese character writing requires fine visuospatial processing and radical-level analysis (J. Yang, 2022). Beginner learners from non-character backgrounds often treat characters as unanalyzable pictures rather than combinable semantic components, leading to frequent errors such as radical misuse, stroke omission, and structural misconfiguration (J. Yang, 2018; X. Yan & Lin, 2023). Whether WL, as a metacognitive tool, can effectively scaffold this specific orthographic challenge remains empirically untested. Does this specificity of the writing system reshape the cognitive pathways of feedback processing? How applicable is WL in CSL writing? These questions lack empirical answers.

Second, learner population characteristics. Secondary school learners in the UK (aged 14–15) represent a distinct learner profile with different cognitive and motivational characteristics compared to university-aged adults. Adolescents are undergoing significant cognitive and metacognitive development but have limited processing capacity and attention spans (Muñoz, 2014). Their learning is also constrained by institutional time limits—typically no more than 2.5 h of Chinese per week and minimal out-of-class input (Tinsley & Board, 2017; Collen, 2023). The feasibility, effectiveness, and learner acceptance of WL in this authentic classroom context remain to be empirically examined. Recent work by Rahim and Yang (2024) has begun to explore languaging with low-proficiency L2 learners, but their study focused on English as the target language and did not address the unique challenges of logographic writing systems. Peng (2024) further emphasized that individual and collaborative feedback processing can yield different developmental trajectories, suggesting that the social and cognitive contexts of feedback matter profoundly for adolescent learners.

From the perspective of the “Intelligent Language Learner,” this study is positioned not as an AI-intervention study, but as an essential foundational investigation into the cognitive and metacognitive mechanisms that constitute intelligent L2 learning. By examining how WL, a form of self-regulated, metacognitive activity, enhances feedback processing, we aim to delineate the cognitive architecture that underpins successful error correction and long-term linguistic development. In this study, the “intelligent language learner” refers to a learner who actively engages in self-regulated, metacognitive processing of linguistic input and feedback—specifically, the capacity to notice gaps in one’s own knowledge, test hypotheses about language forms, and engage in metalinguistic reflection. “Intelligent L2 learning,” accordingly, is understood not as a fixed aptitude or the mere application of learning strategies, but as a dynamic process in which learners monitor their own comprehension and production, allocate attentional resources to formal features of the target language, and strategically use language as a tool for thinking and reflection. WL externalises these internal cognitive processes through written articulation, making them available for conscious inspection and refinement, and thus serves as a key mechanism for fostering intelligent L2 learning. Understanding these mechanisms is paramount for the future development of AI tools. Current AWE systems excel at error detection and providing corrections, but they are often designed as “black boxes” that do not explicitly engage the user’s cognitive architecture (H. Chen & Pan, 2022). Research on learner interaction with AI-generated feedback (H. Yang et al., 2024) has shown that students’ responses to automated feedback often remain at the mechanical, surface level in initial stages, mirroring the very “shallow processing” problem that WL is designed to address. D. Yan and Zhang (2024) found that cognitive engagement with ChatGPT-generated feedback is elicited through “heightened attention to linguistic form, reflective problem-solving, and critical appraisal of AI-generated feedback” precisely the kinds of metacognitive processes that WL explicitly cultivates. By illuminating the hierarchical, attention-driven nature of WL and the individual differences that moderate its effects, this study identifies cognitive-psychological principles—specifically, a noticing → languaging → internalisation processing sequence operationalised through WL and measured via writing accuracy indicators—that could guide the future design of adaptive AI systems. Such systems might be engineered to prompt for, assess, and respond to learners’ metalinguistic reasoning, but these design proposals are theoretical extrapolations from our human-feedback data and require direct empirical testing in AI-mediated contexts. We interpret our accuracy and languaging data as behavioural proxies for this architecture, recognising that cognitive processes are inferred rather than directly observed.

To fill the identified gaps, this study conducted a within-subjects crossover experiment in a beginner CSL classroom in a UK secondary school, investigating the effects of WL with direct feedback on writing accuracy in an authentic teaching context. The study used the General Certificate of Secondary Education (GCSE) writing assessment criteria as a framework for overall performance, while simultaneously measuring writing accuracy from character-level, text-level, and clause-level dimensions to more comprehensively reflect the linguistic performance characteristics of beginner CSL learners. Specifically, the study addressed the following three research questions:

1. Does adding WL to direct feedback produce an immediate, measurable improvement in writing accuracy among beginner CSL learners? Is this effect consistent across orthographic (character accuracy rate), text-level (errors per 100 characters), and syntactic (proportion of error-free clauses) measures?
2. Does learners’ overall writing performance (as measured by GCSE writing scores) change significantly from pre-test to post-test?
3. How do learners perceive direct feedback and WL activities? What are their subjective evaluations of acceptance, understanding, and the role of these activities in writing development?

An intelligent system, whether a human teacher or an AI tool, must be able to adapt its scaffolding based on the learner’s current cognitive state. Our findings operationalize this cognitive state by illuminating the hierarchical nature of feedback processing and the critical role of metacognitive languaging, providing empirical evidence for a cognitive architecture—specifically, a noticing → languaging → internalisation sequence—that could inform the design of AI systems that might adaptively prompt for, assess, and respond to learners’ metalinguistic reasoning. However, as this study did not involve any AI system, these implications are theoretical and await empirical validation in AI-mediated learning environments. These principles, hierarchical targeting, contingent elaboration, adaptive load management, and metacognitive transparency collectively specify the functional requirements for an AI system that genuinely cultivates, rather than circumvents, the learner’s cognitive architecture. This study extends the applicability of the languaging construct to the CSL context and adolescent learner population, providing new evidence from a non-Indo-European language on the interaction between orthographic features and feedback processing. At the same time, the study aims to offer an operational, low-cost feedback-enhancing strategy for overseas secondary Chinese classrooms with limited instructional time.

## 2. Literature Review

Whether direct feedback can effectively promote second language (L2) writing accuracy depends not only on the form of the feedback itself, but also on the depth of cognitive processing that learners apply to the feedback information. From the perspective of cognitive architectures for intelligent L2 learning, the crucial question is not simply *what* feedback is provided, but *how* the learner’s cognitive system perceives, processes, and integrates that feedback into existing knowledge representations. Existing studies, while confirming the conditional effectiveness of feedback, have also exposed the common problem of surface processing: learners often make immediate revisions based on the correct forms provided by the teacher, but repeat the same errors in subsequent writing. This contradiction has led researchers to shift attention from “what feedback provides” to “what happens after feedback” that is, the cognitive and metacognitive processes that mediate between feedback reception and durable learning. “Languaging” has emerged as an important theoretical concept in this shift, offering a window into the cognitive architecture of the intelligent L2 learner.

This literature review first examines the effectiveness of written corrective feedback and the persistent problem of shallow cognitive processing. It then introduces languaging as a theoretical pathway for deepening feedback processing, situating it within the broader framework of cognitive architectures for L2 learning. Finally, it reviews the empirical evidence for WL combined with corrective feedback, identifies critical gaps in the literature particularly concerning typologically distinct target languages, adolescent learner populations, and the implications for AI-assisted learning environments and articulates how the present study addresses these gaps.

### 2.1. The Effectiveness of Written Corrective Feedback: Consensus and Open Questions

Written corrective feedback (WCF) is one of the most extensively researched topics in L2 writing studies. Over the past two decades, debates surrounding the effectiveness of WCF have constituted a major thread in the field, evolving from a binary “to correct or not to correct” to a more nuanced exploration of “what works for whom under what conditions” (Ferris, 1999, 2004; Truscott, 1996). This evolution reflects a growing recognition that feedback effects are not uniform but are mediated by complex interactions among feedback characteristics, learner attributes, and contextual factors a key consideration for understanding the cognitive architectures that underpin intelligent L2 learning.

Truscott (1996), in his influential critique, argued that grammar correction not only fails to promote long-term development but may also increase anxiety and negatively affect writing fluency. This view triggered extensive discussion and ultimately redirected research focus toward more fine-grained investigations of feedback typologies and their differential effects. Ellis (2009) proposed a relatively comprehensive taxonomy, distinguishing direct, indirect, and metalinguistic feedback, among others. On this basis, numerous empirical studies examined the differential effects of feedback types on writing accuracy. Van Beuningen et al. (2008) found that direct feedback was more effective than indirect feedback in improving learners’ writing accuracy in the short term; Bitchener and Knoch (2008) further showed that focused feedback was more effective than unfocused feedback in promoting the acquisition of specific grammatical structures. More recently, a growing body of research has begun to examine the cognitive processes that mediate the relationship between feedback provision and learning outcomes (S. Chen et al., 2016; Storch & Wigglesworth, 2010; Bitchener & Storch, 2016), reflecting a broader shift in the field toward process-oriented investigations of feedback effectiveness.

The past two decades have witnessed a proliferation of empirical studies and meta-analyses that have systematically examined the conditions under which WCF is most effective. In recent years, several meta-analyses have provided more systematic evidence for WCF effects. Kang and Han’s (2015) meta-analysis found that WCF had a significant positive effect on writing accuracy, but the effect was moderated by feedback type, learner proficiency, and task conditions. S. Li and Vuono’s (2019) systematic review reached similar conclusions, underscoring that effect sizes and directions depend on specific implementation conditions. Brown et al. (2023), employing a Bayesian meta-analytic approach, provided further nuance by demonstrating that the effectiveness of WCF is contingent upon how it is delivered and processed, with direct feedback showing larger effects than indirect feedback in the short term but not necessarily in the long term. More critically, for the cognitive perspective advanced in this study, Manchón et al. (2020) found that feedback processing depth was a stronger predictor of learning outcomes than feedback type itself, suggesting that individual differences in cognitive engagement may be more consequential than the structural features of the feedback. These meta-analyses have largely settled the fundamental doubts raised by Truscott (1996), but they also indicate that the cognitive mechanisms through which feedback exerts its effects remain underspecified.

Among feedback types, direct feedback has received particular attention due to its clarity and efficiency. Direct feedback refers to the teacher explicitly identifying errors in the learner’s text and providing the correct linguistic form (Ferris, 2010). For lower-proficiency learners, direct feedback reduces the cognitive load during revision, allowing learners to make corrections without having to infer the correct form themselves (Ferris, 2002). Direct feedback serves a crucial scaffolding function: it reduces the attentional demands of error identification, freeing up cognitive resources that can potentially be redirected toward deeper processing of the correction itself. In CSL teaching, this advantage is especially prominent—beginner learners face not only lexical and syntactic difficulties but also the additional cognitive demands of the Chinese character writing system, which requires fine-grained visuospatial analysis and radical-level processing (J. Yang, 2018, 2022; X. Yan & Lin, 2023).

However, existing studies have also revealed a notable phenomenon: although learners can make immediate revisions based on the correct forms provided by the teacher, the same errors often reappear in subsequent writing. Ferris (2004) pointed out that the long-term effect of feedback depends not on what the teacher provides, but on how the learner processes the feedback. This finding suggests that merely discussing the differential effects of feedback forms is insufficient to fully explain the mechanisms of writing accuracy development. Researchers need to pay further attention to the cognitive processing that occurs *after* feedback namely, whether and how learners deeply process feedback information and transform it into transferable linguistic knowledge. This missing or insufficient cognitive processing constitutes a critical gap in current feedback research, one that is particularly consequential in the CSL context, where character-level errors often involve stroke omission or radical misplacement, requiring cognitive processing fundamentally different from the morphosyntactic errors common in alphabetic L2 contexts (J. Yang, 2018; X. Yan & Lin, 2023).

### 2.2. Languaging: A Theoretical Pathway for Deepening Feedback Processing

The concept of “languaging” is grounded in Vygotsky’s (1978) sociocultural theory, which holds that human cognitive development is mediated through social interaction, and language itself is the most important mediating tool (Lantolf & Thorne, 2006). From this perspective, languaging is not merely a communicative act but a cognitive one a process through which learners use language to organize, structure, and reconstruct their understanding of linguistic knowledge. Swain (2006) defined languaging as “the process of making meaning and shaping knowledge and experience through language,” emphasizing that learners use language to reflect on and reason about language use, thereby promoting attention to and deeper processing of linguistic forms. In the context of intelligent L2 learning, languaging can be understood as a central component of the learner’s metacognitive architecture the set of cognitive processes that enable self-regulation, reflection, and strategic control over learning (Oxford, 2017; Ishikawa & Suzuki, 2023).

Swain and Lapkin (1995) further elaborated on the functions of output, providing a functional explanation for languaging’s benefits. They posited that output serves at least three functions that are directly activated by languaging: the noticing/triggering function, where learners, in the process of producing language, become aware of gaps between their linguistic abilities and expressive needs; the hypothesis-testing function, where output provides opportunities for learners to test their hypotheses about how the language works; and the metalinguistic function, where output triggers reflection on linguistic forms, facilitating the systematization of language knowledge. These three functions correspond closely to the cognitive processes that characterize intelligent learning: monitoring one’s own knowledge state, generating and testing predictions, and engaging in explicit reasoning about the structure of the target system (Ellis, 2009).

This theoretical perspective complements and extends R. W. Schmidt’s (1990) noticing hypothesis. The noticing hypothesis holds that only input that is noticed by the learner can be absorbed and enter the acquisition system; noticing is a necessary condition for language acquisition. In writing instruction, learners often prioritize meaning expression over linguistic forms, so formal errors are easily overlooked. Direct feedback increases the salience of linguistic forms by juxtaposing correct forms with errors, thereby increasing the likelihood that learners notice the errors. However, the noticing hypothesis also reminds us that brief attention to form is not equivalent to understanding and internalization; without further cognitive processing, learners may remain at the level of “seeing and copying a replacement.” R. Schmidt (2001, p. 4) subsequently clarified that “noticing is not a binary phenomenon but a matter of degree,” and that the quality and duration of attention significantly affect learning outcomes. The more fully and persistently learners attend to linguistic features, the higher the likelihood of internalization.

Languaging serves as the critical bridge that transforms “brief noticing” into “sustained processing.” When learners use language to articulate their reasoning about linguistic forms, they prolong and deepen their attention, transforming a fleeting moment of awareness into an active cognitive operation. This process can be understood as a form of metacognitive engagement the deliberate, conscious reflection on one’s own cognitive processes (Flavell, 1979, p. 908; Winne & Nesbit, 2010). The languaging that learners produce when explaining an error is not merely a record of what they know; it is itself a cognitive act that reshapes what they know. By externalizing their reasoning, learners make their implicit, often incomplete intuitions about language available for conscious inspection, revision, and consolidation.

Languaging can be oral or written. Compared with oral languaging, WL in which learners record in writing their explanations and reasoning about their revision actions—has unique advantages that make it particularly suitable for investigating cognitive architecture: it provides more processing time, allows more deliberate and structured reflection, and the written record facilitates later review and retrieval (Ishikawa, 2013, p. 222; Suzuki, 2012, p. 1113). These advantages are especially important for learners at the beginner level, who may need more time to process and reorganize feedback information, and for adolescent learners, whose working memory capacity is still developing (Muñoz, 2014). Moreover, the written artifact of WL offers a unique opportunity for AI-assisted learning. Recent advances in Natural Language Processing (NLP) make it increasingly feasible to analyze learners’ languaging texts in real time not merely for lexical matching, but for metacognitive depth and explanatory accuracy (D. Yan & Zhang, 2024; Klimova & Pikhart, 2023). This technological affordance opens the door for AI systems that can: detect when a learner’s languaging is superficial or inaccurate, provide contingent, real-time follow-up prompts to deepen reasoning, and dynamically adjust the focus of languaging prompts (e.g., from character-level to syntax-level) based on the learner’s performance trajectory. However, for these affordances to be pedagogically effective, they must be guided by an empirical model of how feedback processing unfolds hierarchically a gap that the present study addresses.

However, existing theoretical discussions and empirical studies have been predominantly based on Indo-European languages and adult learners. Whether the special characteristics of Chinese as a target language (non-alphabetic script, the visuospatial burden of orthographic processing) and the cognitive developmental features of adolescent learners affect the effectiveness and mechanisms of languaging strategies remains insufficiently theorized and empirically tested. A scoping review of languaging research in L2 education (C. Li et al., 2023) confirms that the vast majority of empirical studies have been conducted in English-as-a-Foreign-Language (EFL) contexts with university-level participants, leaving critical gaps in our understanding of how languaging functions across different linguistic typologies and learner populations. This is the core question that the present study attempts to address, with particular attention to the implications for understanding the cognitive architectures of intelligent L2 learning. Critically, the written artifact of WL offers a unique opportunity for AI-assisted learning. From the perspective of building intelligent systems, learners’ WL texts provide a rich, ecologically valid data source for assessing their cognitive states and tracking the development of their metalinguistic awareness. Recent advances in natural language processing make it increasingly feasible to analyze the complexity and accuracy of learners’ languaging in real time D. Yan and Zhang (2024), paving the way for AI systems that can adapt their feedback not only to the product (the errors) but also to the process (the learner’s reasoning).

### 2.3. Empirical Evidence for Written Languaging Combined with Corrective Feedback

Empirical studies that place WL within a corrective feedback processing framework are limited in number, but they have initially validated its effectiveness and begun to illuminate the cognitive mechanisms involved. Suzuki (2012, p. 1115), in an early foundational study, asked 24 Japanese EFL learners to write in their L1 (Japanese) explanations of errors that had been directly corrected in their essays. The study found that learners who engaged in WL corrected more errors in immediate revision compared to a control condition that received direct feedback without languaging. Suzuki’s (2012, p. 1115) study also revealed that the *quality* of languaging the depth and accuracy of the explanations provided, was positively correlated with revision success, a finding that has important implications for understanding the cognitive architecture of feedback processing. However, the study’s main focus was on immediate revision rather than long-term retention or transfer to new writing tasks, and the control condition was relatively limited, so the additional benefit of languaging required further controlled testing.

Subsequent studies have gradually supplemented this evidence chain. Yılmaz (2016) found a positive correlation between the quality of WL and successful error correction, suggesting that learners who articulated more accurate and detailed explanations were more likely to internalize the corrections. Suzuki (2017, p. 470) further investigated the relationship between languaging quality and accuracy gains, finding that the production of “high-quality” languaging characterized by accurate metalinguistic explanations—was associated with larger gains in accuracy, whereas “low-quality” languaging showed weaker associations. These findings suggest that the cognitive benefits of WL are not automatic; they depend on the learner’s ability to engage in meaningful metacognitive reflection, which itself may be moderated by individual differences in language aptitude, metalinguistic awareness, and working memory capacity (Ishikawa & Suzuki, 2023). This has direct implications for intelligent feedback systems, which could be designed to not only prompt languaging but also assess its quality and intervene when learners produce superficial or inaccurate explanations (D. Yan & Zhang, 2024).

Moradian et al. (2017), using an improved design with both immediate revision and delayed post-tests, found that WL significantly increased the learning efficiency of corrective feedback. However, that study focused on immediate revision effects and used relatively simple writing tasks, limiting the generalizability of its findings to more complex writing contexts. Niu and You (2020) explored the effects of WL based on indirect corrective feedback on L2 writing accuracy, finding that while accuracy improved significantly in both conditions, the between-group difference did not reach significance, suggesting that the effect of WL may be moderated by feedback type. This moderation effect underscores the importance of understanding how WL interacts with different types of feedback within the learner’s cognitive architecture: with indirect feedback, learners must first identify the error before they can language about it, potentially imposing an additional cognitive load that weakens the added benefit of WL.

In addition, some studies have begun to examine differential effects of WL across task conditions and learner characteristics. Fukuta et al. (2019), extending their research to new text writing on the basis of “indirect feedback plus WL,” found that WL was not superior to other treatments under all conditions, suggesting that its effects may be influenced by learners’ proficiency and feedback type. This finding reminds researchers that the effects of WL may not be universally applicable but are moderated by multiple factors a pattern consistent with the broader literature on individual differences in L2 learning (Skehan, 2015; Oxford, 2017). More critically, recent research has begun to examine how individual differences in cognitive and affective factors including language aptitude, working memory, and motivation moderate the effectiveness of languaging interventions. Peng (2024) found that individual and collaborative feedback processing yielded different developmental trajectories, suggesting that the social and cognitive contexts of feedback processing matter profoundly for adolescent learners. Rahim and Yang (2024) explored languaging with low-proficiency EFL learners and found that while WL was beneficial for some learners, others struggled to engage meaningfully, highlighting the need for adaptive scaffolding a finding with direct relevance to the design of intelligent, AI-powered feedback systems.

Taken together, the available empirical evidence tentatively supports the enhancing effect of WL on feedback processing, but the generalizability of the findings is clearly limited in several critical respects.

First, target language typology. All the above studies were conducted with Indo-European languages (mainly English), and none has tested WL in a CSL teaching context. The Chinese writing system (characters) poses cognitive challenges in orthographic processing that are fundamentally different from those of alphabetic scripts (J. Yang, 2022; X. Yan & Lin, 2023). While errors in alphabetic L2 writing typically involve morphosyntactic features such as verb tense, subject-verb agreement, or article use, errors in CSL writing frequently involve visuospatial features such as radical misplacement, stroke omission, structural disorganization, or confusion between visually similar characters. These different error types engage different cognitive processes and thus may respond differently to WL as a metacognitive intervention. It remains an open empirical question whether WL, as a form of verbalized metalinguistic reflection, can effectively support orthographic error correction, which may require more implicit, procedural learning through repeated visual exposure and motor practice rather than explicit verbal explanation (Chan et al., 2021; Shen & Ke, 2007).

Second, learner population characteristics. All previous studies were conducted with adult or university-level L2 learner populations with more developed cognitive control, greater metalinguistic awareness, and higher levels of autonomous learning ability compared to adolescents (Muñoz, 2014). The cognitive architecture of adolescent learners differs from that of adults in several important ways: their working memory capacity is still developing, their metacognitive skills are less mature, and their motivation and engagement are more variable and context-dependent (Pfenninger & Singleton, 2017). Additionally, the institutional constraints of secondary education limited instructional time, fixed curricula, and examination pressures—pose practical challenges that may affect the feasibility and effectiveness of WL as an instructional strategy. None of the existing studies has examined the effects of WL among adolescent L2 learners in an authentic classroom context.

Third, transfer and delayed effects. Most studies focused on immediate revision effects, with limited investigation of whether intervention effects transfer to subsequent new writing tasks and transfer is precisely a key indicator of whether an instructional intervention promotes durable learning and a robust cognitive architecture. While Suzuki (2012) and Moradian et al. (2017) included some measure of transfer, the time intervals were relatively short, and the transfer tasks were often closely related to the original tasks. Few studies have examined whether the benefits of WL persist over longer time periods or transfer to writing tasks that differ substantially in content and structure from the training tasks.

Fourth, the development of AI-mediated learning tools has largely bypassed the cognitive mechanisms of feedback processing. Most commercial AWE platforms (e.g., Grammarly, Criterion, Pigai) and generative AI writing assistants (e.g., ChatGPT-based tutors) provide corrections with minimal or no scaffolding for learners’ metacognitive elaboration (Zhang & Hyland, 2022; Barrot, 2023). The implicit pedagogical assumption that providing the correct form is sufficient for learning is precisely the “shallow processing” pitfall that WL is designed to overcome (Ranalli, 2018). While studies have begun to examine learners’ cognitive engagement with AI-generated feedback (D. Yan & Zhang, 2024), they have not systematically operationalized what constitutes deep processing or how AI systems might elicit it. The hierarchical, individual-difference-moderated cognitive architecture revealed in the present study fills this lacuna by providing empirically grounded design parameters for adaptive AI feedback systems. These gaps constitute the basic positioning of the present study: using a within-subjects crossover design in an authentic UK secondary school beginner CSL classroom, we examine the immediate and cross-task transfer effects of WL on writing accuracy, while also revealing learners’ cognitive characteristics during feedback processing through qualitative analysis of their languaging texts and interviews. By investigating an under-researched learner population (adolescent CSL beginners) and a typologically distinct target language (Chinese), this study aims to extend the languaging construct beyond the boundaries of previous research and contribute to a more robust, context-sensitive theory of how intelligent L2 learners process feedback. Furthermore, by identifying the specific cognitive processes engaged during WL attention to characters, vocabulary, syntax, and metalinguistic reflection this study provides foundational insights that can inform the design of intelligent feedback systems capable of scaffolding metacognitive engagement. In this way, the present research contributes not only to the pedagogical literature on CSL writing instruction but also to the broader interdisciplinary conversation on the cognitive architectures that enable intelligent L2 learning in an increasingly technology-mediated educational landscape.

## 3. Theoretical Framework

This study is underpinned by three interrelated theoretical perspectives—sociocultural theory (Vygotsky, 1978), the output hypothesis (Swain, 1985), and the noticing hypothesis (R. W. Schmidt, 1990) which together form a progressive explanatory chain for understanding the cognitive architecture of intelligent L2 learning. Each theory addresses a different level of analysis: sociocultural theory explains *why* language mediates cognition; the output hypothesis explains *what* kinds of language activity promote acquisition; and the noticing hypothesis explains *how* attention translates into internalization. Together, they provide a comprehensive framework for understanding how WL functions as a metacognitive tool that deepens feedback processing and fosters durable L2 writing development. This integrated framework not only guides the design of the present study but also offers theoretical grounding for the development of intelligent, AI-powered feedback systems that aim to scaffold metacognitive engagement.

### 3.1. Sociocultural Theory: Language as a Mediating Tool for Cognition

Sociocultural theory, rooted in the work of Vygotsky (1978), provides the most fundamental theoretical premise for this study. Its core tenet is that human cognitive development is mediated through social interaction and culturally constructed tools, among which language is the most important (Lantolf & Thorne, 2006). From this perspective, language is not merely a vehicle for communication; it is a psychological tool that fundamentally shapes how we think, reason, and learn. When learners use language to organize their thinking whether through speaking or writing language ceases to be merely a tool for expression and becomes part of the thinking process itself.

This perspective has profound implications for understanding the cognitive architecture of intelligent L2 learners. In Vygotsky’s (1986) framework, higher psychological functions (such as voluntary attention, logical memory, and conceptual thinking) originate in social interaction and are gradually internalized through the mediation of language. This process of internalization is not a passive transfer of external knowledge to internal storage; it is an active, constructive process in which learners use language to transform external social activities into internal cognitive resources. In the context of feedback processing, when a learner writes down an explanation such as ‘I wrote 大 (big) instead of 太 (too) because I forgot the dot,’ they are engaging in a form of self-directed dialogue that externalizes internal cognitive processes, making them available for conscious inspection, revision, and consolidation.

In this study, the WL texts are precisely the material records of this “language-mediating-cognition” process. Learners organize, structure, and reconstruct their understanding of language rules through writing. This process transforms implicit, often vague intuitions about language into explicit, inspectable reasoning. Critically, the written artifact created through WL is not merely a record of what the learner already knows; it is itself a tool that reshapes cognition. By rendering thinking visible, the written text allows learners to examine their own reasoning, identify gaps or inconsistencies, and refine their understanding a form of self-scaffolding that is central to intelligent, autonomous learning (Oxford, 2017).

From the perspective of AI-assisted learning, sociocultural theory also highlights the potential of intelligent systems to serve as mediating tools that interact with learners’ cognitive processes. An AI writing assistant, for example, is not merely a source of feedback but a sociocultural artifact that can mediate and potentially transform the learner’s cognitive activity. However, as Godwin-Jones (2022) cautions, the design of such tools must be guided by an understanding of how learners actually use language as a mediational tool, rather than by technological affordances alone. This study contributes to that understanding by illuminating how WL, as a form of language-mediated cognition, operates in a specific L2 learning context.

### 3.2. The Output Hypothesis: The Functions of Output in L2 Acquisition

The output hypothesis, proposed by Swain (1985) and elaborated in subsequent work (Swain & Lapkin, 1995; Swain, 2006), provides the functional mechanism that explains why languaging promotes acquisition. Swain developed the output hypothesis through long-term observation of Canadian French immersion programs, where she found that learners’ grammatical accuracy did not reach native-speaker levels despite abundant comprehensible input (Krashen, 1985). This finding led her to propose that output the production of language plays an independent and critical role in acquisition that is not fulfilled by input alone.

Swain and Lapkin (1995) identified three functions of output, noticing/triggering, hypothesis-testing, and metalinguistic reflection, each of which is directly activated by the WL task in this study (for a full theoretical discussion, see Section 2.2). In essence, the WL task operationalises these functions by requiring learners to explicitly identify errors (noticing), propose and justify corrections (hypothesis-testing), and articulate underlying linguistic rules (metalinguistic reflection), thereby transforming output from a display of known forms into an exploration of unknown ones.

Building on this foundation, Swain (2006) introduced the concept of “languaging” to capture the cognitive processes through which learners use language to mediate their own learning. Languaging is the externalization of cognitive processes through language, making thinking, reasoning, and rule formation observable and amenable to repeated processing. Importantly, not all output is equally effective; only output that compels learners to notice gaps, test hypotheses, and engage in metalinguistic reflection truly promotes language development. WL, as operationalized in this study, satisfies all three conditions by requiring learners to explicitly identify errors, explain reasons for corrections, and reflect on language rules, thereby transforming output from “displaying what is known” to “exploring what is unknown.”

The output hypothesis illuminates how WL enhances the learner’s metacognitive capacity. By systematically externalizing internal reasoning, WL reduces cognitive load, making more cognitive resources available for analysis and consolidation. This is particularly important for beginner learners, whose working memory is heavily burdened by the demands of basic language production. For AI-assisted learning environments, the output hypothesis suggests that intelligent systems should be designed not merely to provide corrective input but to actively elicit output, and specifically, the kind of reflective, metalinguistic output that WL embodies (D. Yan & Zhang, 2024).

### 3.3. The Noticing Hypothesis: Attention as a Necessary Condition for Acquisition

The noticing hypothesis, advanced by R. W. Schmidt (1990) and R. Schmidt (2001), further specifies how cognitive processing occurs at the level of attention, providing a more granular account of the micro-processes involved in languaging. R. W. Schmidt (1990) argued that only input that is noticed by the learner can be absorbed and enter the acquisition system; noticing is a necessary condition for language acquisition. This hypothesis is rooted in the observation that learning cannot occur without awareness of what is being learned a principle that has been supported by decades of research on attention and memory.

R. Schmidt (2001, p. 4) subsequently refined the noticing hypothesis in several important ways. He distinguished between different levels of awareness, noting that noticing is not a binary phenomenon (noticed vs. unnoticed) but a continuum ranging from detection (the registration of stimuli) to awareness (conscious recognition) to understanding (the insight into underlying rules). He also emphasized that noticing is not automatic; its degree and duration significantly affect learning outcomes. The more fully and persistently learners attend to linguistic features, the higher the likelihood of internalization. Critically, Schmidt argued that the “depth” of processing matters: shallow attention to surface features leads to shallow learning, while deeper, more elaborate processing leads to more durable learning.

In writing instruction, learners often prioritize meaning expression over attention to linguistic form, so formal errors are easily overlooked or processed superficially. Direct feedback increases the salience of linguistic forms by juxtaposing correct forms with errors, thereby triggering initial noticing. However, as Ferris (2004) and others have noted, brief attention to form is not equivalent to understanding and internalization. Without further cognitive processing, learners may remain at the level of “seeing and copying a replacement” a surface-level strategy that does not lead to durable learning.

WL plays a critical role precisely at this point: it deepens and extends the initial noticing triggered by feedback, transforming brief attention into sustained, operational cognitive processing. When learners are required to articulate their revision reasoning in writing, they cannot simply copy the correct form; they must attend to the nature of the error, the structure of the correct form, and the relationship between the two. This process prolongs attention, deepens processing, and promotes the kind of elaborative rehearsal that strengthens memory representations (Craik & Lockhart, 1972). The written record of this process also allows for subsequent review, providing additional opportunities for retrieval and consolidation.

The noticing hypothesis underscores the critical role of attentional control in intelligent L2 learning. The intelligent learner is not merely one who receives more input or feedback, but one who actively allocates attention to relevant features and sustains that attention long enough for learning to occur. WL can be understood as a metacognitive strategy that enhances attentional control by structuring and externalizing the learner’s focus of attention, thereby promoting deeper cognitive processing (Oxford, 2017; Ishikawa & Suzuki, 2023).

In the context of AI-assisted learning, the noticing hypothesis has direct implications for system design. Intelligent feedback systems should be designed not only to *present* corrections but also to *guide* attention and *sustain* engagement. Research on human–AI interaction in L2 learning (Klimova & Pikhart, 2023; D. Yan & Zhang, 2024) suggests that learners often process AI-generated feedback superficially, replicating the very “shallow processing” problem observed with human feedback. To address this, intelligent systems could be designed to prompt learners to articulate their understanding of corrections, thereby deepening attention and promoting the elaborative processing that the noticing hypothesis identifies as critical for acquisition.

### 3.4. An Integrated Cognitive Architecture: From Theory to Operationalization

At the operational level, these three theories jointly determine the key design choices and analytical approaches of this study. They are not merely parallel perspectives but form an integrated, multilevel account of how WL contributes to intelligent L2 learning.

The WL task was guided by three questions (“What is wrong here?”, “How should it be corrected?”, and “Why should it be changed this way?”), which were explicitly designed to activate the three functions described by Swain and Lapkin (1995). The noticing hypothesis provided the rationale for using direct feedback rather than indirect feedback: direct feedback ensures that even learners with limited proficiency can identify the target errors, thereby initiating the noticing process that WL then elaborates. Sociocultural theory explains why the WL task is effective: it provides learners with a mediating tool (written language) that externalizes internal cognitive processes, making them available for conscious inspection and manipulation.

The choice of three layered indicators character accuracy rate, errors per 100 characters, and proportion of error-free clauses corresponds to the different linguistic levels at which learner attention may dwell (character forms, overall error density, and syntactic structure). Their combined use can sensitively capture differences in cognitive processing outcomes at different levels, reflecting the hierarchical nature of the cognitive architecture that WL engages. As argued earlier, WL’s effects are expected to manifest first at lower levels (character and word accuracy) before transferring to higher-level syntactic accuracy a prediction consistent with both cognitive load theory (Sweller, 1988) and the hierarchical nature of language processing. Specifically, the hierarchy we hypothesise is grounded in the following theoretical rationale: character-level processing requires relatively discrete visuospatial operations and imposes the least cognitive load; vocabulary-level processing involves semantic selection and collocational constraints and imposes moderate cognitive load; clause-level processing requires the simultaneous integration of orthographic, lexical, syntactic, and discourse-level information and imposes the highest cognitive load. This progression from low to high cognitive load corresponds to a progression from local to global processing, a well-documented pattern in both L2 acquisition and cognitive skill acquisition (Anderson, 1983; DeKeyser, 2020; Skehan, 2015). Accordingly, we predict that the facilitative effects of WL will be strongest at the lowest level (error density, which captures errors across all levels), weaker at the intermediate level (character accuracy), and weakest or absent at the highest level (proportion of error-free clauses) in the short term, with longer-term effects potentially cascading upward as lower-level processing becomes increasingly automatised.

This integrated framework thus serves a dual purpose. It not only guides the empirical design of this study but also provides a functional specification for intelligent feedback systems. A truly ‘intelligent’ tool should not merely present corrections but should be engineered to elicit and amplify the very cognitive processes noticing, hypothesis-testing, and metalinguistic analysis that our framework identifies as central to the learner’s cognitive architecture. The theoretical framework thus bridges the gap between cognitive architecture research and AI pedagogy, offering principles that can guide the development of more intelligent, human-centered learning technologies.

In summary, this study’s theoretical framework offers a coherent account of the cognitive architecture of intelligent L2 learning, integrating sociocultural, output-based, and attentional mechanisms to explain how WL functions as a metacognitive amplifier. By empirically testing the predictions of this framework in a novel context (adolescent CSL learners in an authentic classroom), the study contributes both to theoretical understanding and to the practical design of interventions human and AI-mediated that can foster intelligent language learning.

In this study, the term ‘cognitive architecture’ refers specifically to the functional organisation of metacognitive processes through which learners perceive, process, and internalise written corrective feedback. This architecture is not assumed to be a comprehensive model of all cognitive faculties involved in L2 writing, but rather a delineable set of processing stages, noticing, hypothesis-testing, and metalinguistic reflection, that mediate between feedback reception and durable learning. These stages are operationalised through observable indicators: noticing is triggered by the direct feedback itself; hypothesis-testing and metalinguistic reflection are evidenced by the content and quality of learners’ WL texts; and internalisation is inferred from improvements in writing accuracy (errors per 100 characters, character accuracy rate, proportion of error-free clauses) and transfer to subsequent performance (GCSE scores). Thus, our accuracy measures and languaging texts function as behavioural and textual proxies for the underlying cognitive processes, rather than direct measures of cognitive architecture per se. We acknowledge that these proxies are indirect, and we interpret our findings as providing empirical evidence for the operation of this architecture, not as a direct delineation of neurological or comprehensive cognitive structures.

**Figure 1:**
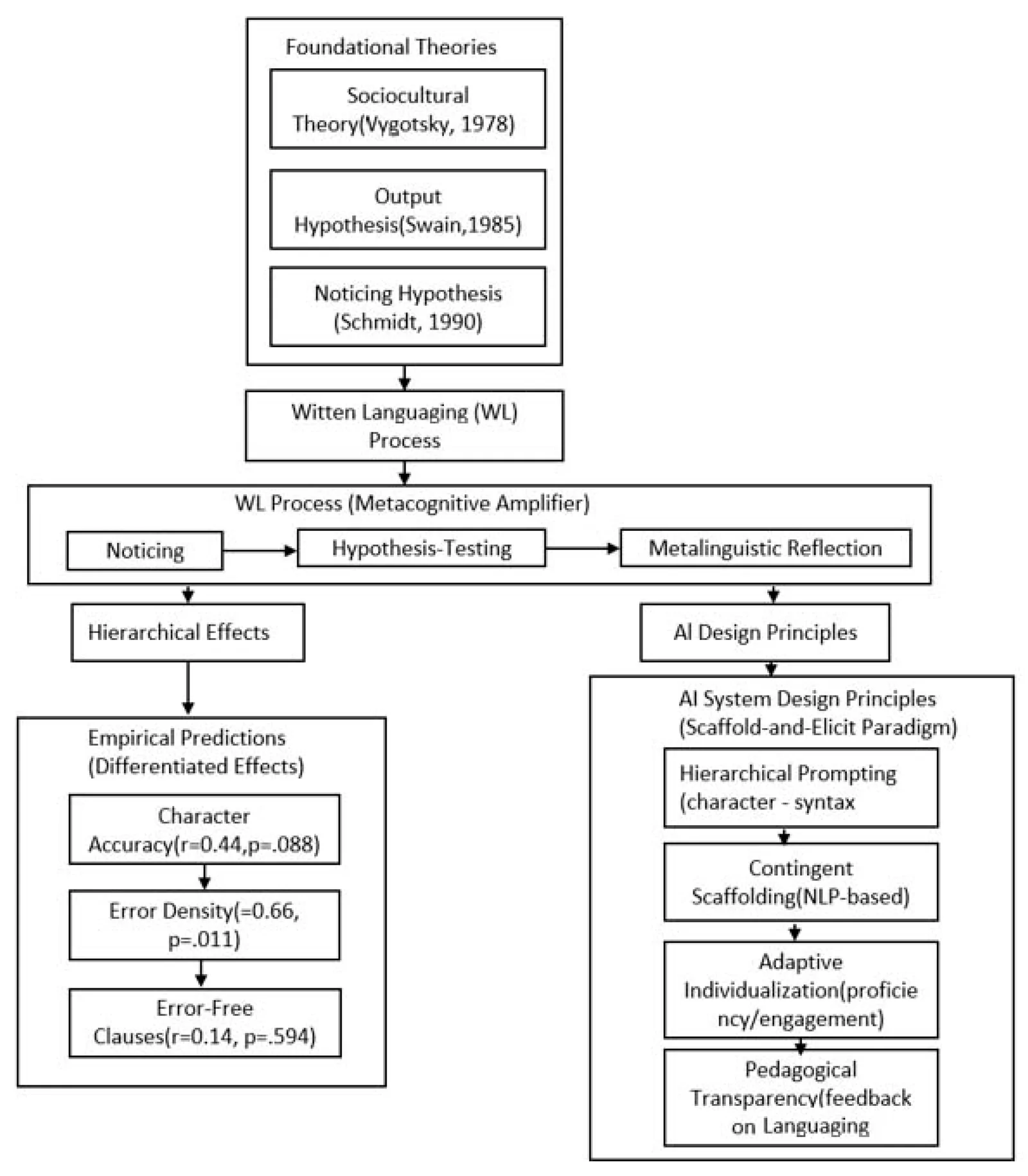
An Integrated Cognitive Architecture for Intelligent L2 Feedback Processing. In this study, this architecture is operationalised as a three-stage processing sequence—noticing (triggered by direct feedback) → hypothesis-testing and metalinguistic reflection (mediated by WL) → consolidation and transfer (evidenced by accuracy gains and subsequent performance). Writing accuracy measures and languaging texts serve as behavioural proxies for the underlying cognitive processes, rather than direct measures of cognitive architecture per se.

Figure 1 presents the integrated theoretical framework that guides this study. The framework illustrates how three foundational theories Sociocultural Theory, the Output Hypothesis, and the noticing hypothesis converge to specify the cognitive architecture of intelligent L2 feedback processing. At the core of this architecture is the WL process, which transforms initial noticing into sustained metacognitive engagement through hypothesis-testing and metalinguistic reflection. This processing is hypothesized to follow a hierarchical pattern, with benefits first manifesting at local linguistic levels before transferring to higher-level syntactic integration. The framework further specifies two key outputs: (1) empirical predictions regarding the differentiated effects of WL across three accuracy indicators, and (2) design principles for AI-powered feedback systems that aim to scaffold, rather than supplant, the learner’s metacognitive activity. This dual-purpose framework thus bridges cognitive architecture research and AI pedagogy.

## 4. Methodology

This study employed a within-subjects crossover design to examine the effects of WL with direct feedback on writing accuracy in an authentic beginner CSL classroom in a UK secondary school. The methodological choices were guided by the theoretical framework outlined above, which conceptualizes WL as a metacognitive tool that engages specific cognitive processes noticing, hypothesis-testing, and metalinguistic reflection within the learner’s cognitive architecture. The design was therefore structured to isolate the cognitive effect of WL from other confounding variables, to capture its effects at multiple linguistic levels, and to investigate individual differences in cognitive engagement.

### 4.1. Research Design

The study used a within-subjects crossover design, in which each participant experienced both experimental conditions in a balanced order: Condition A was “direct feedback + WL”, and Condition B was “direct feedback only” (NOWL). A within-subjects design was chosen over a between-subjects design for two primary reasons, both grounded in the study’s focus on cognitive architecture.

First, control of individual differences. Beginner learners show significant individual differences in language aptitude, orthographic processing ability, working memory capacity, and metacognitive awareness (Muñoz, 2014; Skehan, 2015; Ishikawa & Suzuki, 2023). These cognitive and affective individual differences are central to understanding the architecture of intelligent L2 learning, but they also pose a significant threat to internal validity in small-sample research. Under small-sample conditions, a between-subjects design may confound these individual differences with treatment effects. By using each participant as their own control, the within-subjects design significantly reduces between-subject variability and increases statistical power and internal validity (Sibbald & Roberts, 1998; Wellek & Blettner, 2012). This design choice is therefore not merely a practical convenience but a theoretically motivated decision to isolate the cognitive effect of WL from stable learner characteristics.

Second, crossover control of order effects. The crossover arrangement each participant receiving both conditions in a balanced order controls for order effects such as practice effects, fatigue, or task-specific learning that might otherwise confound the results. By counterbalancing the sequence of conditions across two groups, the design ensures that any observed differences between conditions cannot be attributed to the order in which they were administered. This is particularly important in cognitive research, where practice effects can be substantial.

Participants were assigned to one of two sequence groups through stratified randomization based on pre-test writing scores. Students were ranked by their pre-test GCSE writing total score (out of 16) and paired, then randomly assigned within each pair to ensure high- and low-scoring students were evenly distributed across the two groups. Due to the odd sample size, the two sequence groups differed slightly in number: Sequence Group 1 (*n* = 7) received A → B, and Sequence Group 2 (*n* = 8) received B → A (see Table 1). Under this crossover design, each participant provided one writing performance data point per condition, resulting in 15 × 2 = 30 observational data points for analysis.

To enhance the clarity of the experimental procedure, the following table provides a visual summary of the study timeline, tasks, and conditions (Table 2).

### 4.2. Participants and Research Context

#### 4.2.1. Participants

The initial sample consisted of 18 students enrolled in the GCSE Mandarin Foundation Tier course at a secondary school on the Isle of Wight, UK. All students came from the same intact class, taught by one teacher with five years of Chinese teaching experience. After data completeness screening, three participants were excluded from the final analysis: two produced fewer than 10 Chinese characters across multiple writing tasks (preventing meaningful accuracy analysis), and one had more than 40% missing data due to repeated absences. The final effective sample comprised 15 participants.

The participants’ background characteristics are summarized in Table 3: 7 males (46.7%) and 8 females (53.3%), aged 14–15 years (M = 14.93, SD = 0.26). All participants were native English speakers, had no Chinese family background, had studied Chinese for 2 years, and were at the GCSE Mandarin Foundation Tier stage. According to the *Chinese Language Proficiency Standards for International Chinese Language Education*, their comprehensive proficiency roughly corresponds to Level 1–2 of the elementary stage. In terms of writing ability, participants could complete simple sentences and short texts (approximately 75 characters), but their character writing accuracy, lexical choice appropriateness, and basic syntactic stability remained quite variable.

These participants are characterized by limited working memory capacity for L2 processing, emerging but not yet fully mature metacognitive skills, and a heavy reliance on external scaffolding for error detection and correction (Muñoz, 2014; Pfenninger & Singleton, 2017). Their status as “beginner” learners means that their cognitive resources are heavily taxed by basic language production, leaving limited attentional capacity for self-monitoring and error correction a key rationale for the use of direct feedback and the WL scaffold.

#### 4.2.2. Teaching Context

The study was implemented in the participants’ regular classroom environment; all writing tasks and revision activities were embedded in their normal Chinese curriculum. The school’s GCSE Chinese course followed the AQA examination board syllabus, with three 50-min lessons per week, totalling 2.5 h of classroom contact time per week. The curriculum covered listening, speaking, reading, and writing skills, with writing instruction accounting for about 20% of class time. According to a background questionnaire, over 80% of students reported spending less than 30 min per day on Chinese outside class, and nearly half reported almost no exposure to Chinese beyond homework. This input profile reflects the typical characteristics of Chinese teaching in UK secondary schools: limited instructional time, insufficient out-of-class input, and learning heavily reliant on the classroom environment (Tinsley & Board, 2017; Collen, 2023).

This context is theoretically significant for studying cognitive architecture. The limited input and instructional time mean that learners have few opportunities for implicit, incidental learning; their development depends heavily on explicit instructional interventions that optimize cognitive processing efficiency. WL, as a metacognitive intervention that deepens processing of limited instructional input, is therefore particularly well-suited to this context. The findings of this study thus have direct relevance for resource-constrained teaching environments where maximizing the cognitive yield of limited instructional time is paramount.

During the study period (March to May 2025), all participants were preparing for the GCSE Mandarin Foundation Tier examination. The school used the textbook *GCSE Chinese (AQA)*, with teaching focused on examination topics (e.g., personal information, school life, holiday experiences, eating habits). The writing tasks used in this study were selected or adapted from GCSE Chinese writing authentic examination materials (AQA, 2024), with task structures consistent with students’ regular classroom practice and exam preparation. Each writing task contained four content prompts and required students to write a short text of about 75 Chinese characters within 30 min.

Ethical approval was obtained from the Ethics Review Committee of Northeast Normal University and the school administration. Approval Number: 202602035. Before the study began, researchers provided all participants with detailed information about the research purpose, procedures, data use, and participant rights. All participants signed written informed consent forms, and were informed that participation was entirely voluntary and that they could withdraw at any time. All data were anonymized after collection, with participants identified by alphanumeric codes (S1–S15), and no personally identifiable information was recorded.

### 4.3. Experimental Conditions

This study compared two experimental conditions, both implemented in the same classroom setting, with feedback provided by the same teacher. The design of these conditions was informed by the theoretical framework: Condition A (WL) operationalizes the full cognitive architecture of noticing → languaging → internalization, while Condition B (NOWL) operationalizes noticing without the extended metacognitive processing.

Condition A: Direct Feedback + WL. Participants received direct feedback from the teacher on their first draft. Direct feedback was operationally defined as the teacher explicitly marking errors in the student’s text and providing the correct linguistic form, including corrections for character writing, word choice, and syntactic structure (Ferris, 2010). The feedback itself contained no metalinguistic explanation or systematic grammar instruction; its main function was to enable learners to clearly identify language errors and make corrections—thereby triggering the initial *noticing* that R. W. Schmidt (1990) identified as a prerequisite for acquisition. After receiving feedback, participants were required to complete a WL record sheet, providing written explanations for their revision actions. The record sheet contained three guiding questions: (1) “Why is this language form incorrect/problematic?” (2) “Why did the teacher provide feedback on this form?” (3) “If you cannot explain what the problem is, you may write ‘I don’t know’.” Participants were allowed to respond in their L1 (English) to ensure that Chinese writing ability did not constrain their ability to articulate metalinguistic reasoning. These guiding questions were explicitly designed to activate the three functions of output identified by Swain and Lapkin (1995): noticing (question 1), hypothesis-testing (question 2), and metalinguistic reflection (question 3).

Condition B: Direct Feedback Only (NOWL). Participants received the same direct feedback on their first draft from the teacher and were asked to revise the text based on the feedback, but they were not required to complete a WL record sheet or provide any written explanation. This condition serves as a control that isolates the cognitive effect of WL: any difference between conditions can be attributed to the additional metacognitive processing elicited by the WL task, rather than to the feedback itself.

Under both conditions, the scope and manner of teacher feedback were consistent: the teacher provided direct corrections for all identifiable errors (including character, vocabulary, grammar, and word order errors) without selective feedback. Feedback was marked in red ink directly on the students’ written texts, with correct forms written above or beside the errors. All feedback was provided by the same teacher to ensure consistency in feedback style and standards.

### 4.4. Writing Tasks and Experimental Procedure

The study lasted six weeks, with all activities embedded in the participants’ regular Chinese lessons. Four writing tasks were administered: pre-test, two experimental tasks, and post-test. All tasks were selected or adapted from GCSE Chinese Foundation Tier writing examination materials (AQA, 2024). Each task required participants to write a short text of about 75 Chinese characters within 30 min, with four content prompts. The topics for each task were: pre-test and post-test “holiday experiences” (same topic); Experimental Task 1 “travel plans in China”; Experimental Task 2 “school life”.

The same-topic pre-test/post-test design was chosen to maximize control over task complexity, content familiarity, and information generation load, ensuring that observed differences reflected changes in linguistic forms rather than task difficulty (Van Beuningen et al., 2008). This design choice is particularly important for cognitive research, as differences in task difficulty can affect cognitive load and thus confound measures of accuracy. The pre-test and post-test were separated by three weeks, during which students completed different-topic writing tasks and received feedback under different conditions, reducing the likelihood of direct memory of the original text.

The overall procedure was as follows:

Week 1: Researchers explained the research purpose and procedures to participants, obtained informed consent from participants and their guardians, and confirmed through classroom observation and teacher evaluation that participants’ language proficiency met the definition of beginner learners.

Week 2 (Pre-test): Participants completed the pre-test writing task (holiday topic) without any feedback or revision. No corrective feedback or revision requirement was provided at this stage, to avoid the pre-test itself becoming part of the intervention. The pre-test texts were used for: (1) obtaining overall writing scores according to GCSE criteria (for stratified randomization and pre-post comparison); (2) calculating baseline accuracy indicators.

Week 3 (Experimental Task 1): Participants completed the first experimental writing task (China travel topic). After submission, the teacher provided direct feedback on all identifiable errors in each participant’s text during breaks or after class, and returned the marked essays the next day. Sequence Group 1 then completed the WL record sheet and revised accordingly (Condition A), while Sequence Group 2 revised the text without WL (Condition B). The revision phase was limited to 15 min, completed independently in class.

Week 4 (Experimental Task 2): The second experimental writing task (school life topic) was administered, with treatment conditions crossed over. Sequence Group 1 received Condition B (direct feedback only), and Sequence Group 2 received Condition A (direct feedback + WL). All other procedures were the same as Week 3.

Week 5 (Post-test): Participants completed the post-test writing task, using the same topic as the pre-test (holiday experiences). No feedback was provided at this stage, to isolate the pure effect of the intervention.

Week 6 (Semi-structured interviews): Semi-structured interviews were conducted with 5 voluntarily participating participants to explore their perceptions and attitudes towards feedback and WL activities.

### 4.5. Measurement Indicators

Writing accuracy was operationalized through three complementary indicators. In second language writing research, accuracy is generally understood as the degree of control over target language formal rules and is an important component of the CAF (Complexity-Accuracy-Fluency) framework. However, existing research also points out that writing accuracy is not a unitary construct; different indicators capture different aspects of formal language control (Liu & Shi, 2025). Given that the participants were beginner UK Chinese learners with relatively short writing texts, and considering the linguistic characteristic of Chinese using characters as the basic writing unit, this study selected the following three indicators from the character, text, and clause levels: character accuracy rate, errors per 100 characters, and proportion of error-free clauses.

These three indicators capture different levels of cognitive control in L2 writing:

Character accuracy rate reflects learners’ control over visuospatial orthographic processing the cognitive ability to retrieve and execute correct stroke sequences, radical configurations, and spatial arrangements. This is the most local level of cognitive control and is predicted to be most sensitive to WL, as character errors are discrete and readily identifiable.

Errors per 100 characters provides a standardized overall error density indicator, capturing the cumulative effect of errors across all linguistic levels. It reflects the learner’s general ability to monitor and regulate the accuracy of their output.

Proportion of error-free clauses is the most stringent indicator, reflecting learners’ ability to simultaneously coordinate multiple linguistic dimensions (orthographic, lexical, syntactic) within a single syntactic unit. This requires more integrated cognitive control and is predicted to be less sensitive to short-term WL interventions, consistent with the hierarchical nature of the cognitive architecture hypothesized above.

Character accuracy rate reflects learners’ orthographic accuracy control at the character level. Any character that deviates from the standard form due to missing strokes, extra strokes, radical errors, radical position reversal, or structural disorder was counted as an erroneous character. Characters that were formally standard, correctly structured, and semantically appropriate to the context were counted as correct characters. Each erroneous character was counted as one independent error instance; when the same error appeared multiple times in the same text, it was counted according to the actual number of occurrences. The formula was: Character accuracy rate = (number of correct characters ÷ total number of characters) × 100%.

Errors per 100 characters provides a standardized overall error density indicator, controlling for the influence of text length differences on error count comparisons (Chandler, 2003). All identifiable errors including orthographic, lexical, syntactic, and word-order errors were counted across the entire text. The formula was: Errors per 100 characters = (total number of errors ÷ total number of characters) × 100. In second language writing research, the error rate per 100 words (or characters) is considered one of the most commonly used and reliable indicators of linguistic accuracy, particularly suitable for learners with short texts and unstable language structures.

Proportion of error-free clauses reflects learners’ ability to produce clauses that are both syntactically and orthographically correct. Following established procedures (Wolfe-Quintero et al., 1998; Polio & Shea, 2014), a clause was defined as a syntactic unit centred on a predicate (verbal or adjectival) expressing a relatively complete event or state (Zhao, 1968). In clause counting, the principle of “one predicate corresponds to one clause” was followed. For beginner learners’ Chinese written texts, even if the sentence structure was incomplete or contained formal errors, as long as the predicate component could be identified, it was still counted as a valid clause; nominal phrases, temporal phrases, or locative phrases without predicates were not counted as clauses. In judging error-free clauses, a binary principle was adopted: if any identifiable language error appeared in a clause, that clause was judged as “erroneous”; only if the clause contained no identifiable errors was it counted as “error-free.” Very short expressions (e.g., “hello”), expressions lacking a complete syntactic structure or a predicate centre (e.g., “goodbye”), were not included in the total clause count. The formula was: Proportion of error-free clauses = (number of error-free clauses ÷ total number of clauses) × 100%.

Overall writing performance (GCSE score) was used as a supplementary dependent variable. Pre-test and post-test essays were scored according to the AQA GCSE Mandarin Foundation Tier writing assessment criteria (AQA, 2024). The marking scheme has a total of 16 points, covering two dimensions: Content and Communication (10 points) and Quality of Language and Accuracy (6 points). This assessment tool was chosen because the participants were beginner Chinese learners at the UK secondary level, and their language proficiency, writing task formats, and learning objectives were closely aligned with the GCSE Foundation Tier. Pre-test and post-test essays were independently scored by two raters, with good inter-rater reliability (pre-test r = 0.975, *p* < .001; post-test r = 0.962, *p* < .001).

### 4.6. Qualitative Data Sources and Analysis

Qualitative data came from two sources: WL record sheets and semi-structured interviews. These data were essential for investigating the *process* of cognitive engagement during WL (RQ3) and for understanding how individual differences moderate the effects of WL.

WL record sheets. In Condition A, participants completed WL record sheets containing three guiding questions and blank response areas. Participants were encouraged to “write down your thoughts as detailed as possible, in English, Chinese, or a mix of both.” These records were collected by the researchers after class, transcribed into electronic documents, and analysed as qualitative data. A total of 275 meaning units were collected. From a cognitive research perspective, these texts provide a window into learners’ metacognitive processing their awareness of errors, their reasoning about corrections, and their ability (or inability) to articulate metalinguistic explanations.

Semi-structured interviews. After the intervention, semi-structured interviews were conducted with 5 participants (S1–S5) on a voluntary basis. Interviewees were selected voluntarily, covering different genders and writing proficiency levels. Each interview lasted about 10 min and was conducted face-to-face. The interview guide revolved around three themes: overall perceptions and acceptance of direct feedback and WL activities; self-assessment of the feedback understanding and processing process; subjective evaluation of the role of these activities in writing development. All interviews were audio-recorded with consent and transcribed verbatim, conducted in English. Basic information about the interviewees is shown in Table 4.

Qualitative data analysis. Qualitative data were analysed using thematic content analysis (Schreier, 2012; Graneheim & Lundman, 2004). The analysis proceeded in three phases: first, each WL record was segmented into meaning units—discrete statements expressing a single idea or observation; second, each meaning unit was assigned an initial label using open coding, with coding schemes referencing established categories from error analysis and second language writing research (Ferris, 2002), and refined iteratively during analysis; third, initial codes were grouped into broader core categories through the constant comparative method (Glaser & Strauss, 1967). Interview transcripts were analysed using a combined deductive-inductive approach, with the deductive part guided by the theoretical framework (sociocultural theory, output hypothesis, noticing hypothesis) and inductive coding used to capture participants’ spontaneous expressions. This dual approach ensured that the analysis was both theory-informed and grounded in participant voices.

### 4.7. Data Analysis

Before data analysis, the original writing texts underwent the following pre-processing: text length standardisation for indicators involving error counts, values were converted to relative indicators based on actual text length; error counting using an error-instance counting method, each erroneous character or erroneous form appearing once in the text was counted as one independent error instance; if the same error appeared multiple times in the same text, it was counted according to the actual number of occurrences; error-free clause judgement using a binary principle, any clause containing any identifiable error was judged as “erroneous.”

Quantitative data were analysed using SPSS 26.0. Given the within-subjects design and small sample size (N = 15), the Wilcoxon signed-rank test was selected as the primary inferential statistical method for all paired comparisons. This test was chosen for three reasons: first, the Shapiro–Wilk normality test showed that errors per 100 characters under the NOWL condition significantly deviated from normality (W = 0.586, *p* < .001); second, for a sample size of 15, non-parametric tests are more robust to violations of normality; third, using the same non-parametric test across all analyses maintains analytical consistency (Larson-Hall, 2016; Plonsky & Oswald, 2014). Effect sizes (r) for all significant comparisons were calculated using the formula r = |Z|/√N, where N is the number of valid paired observations. Following conventions in second language research, r values of 0.10, 0.30, and 0.50 were interpreted as small, medium, and large effects, respectively (Plonsky & Oswald, 2014).

To ensure objectivity in scoring, pre-test and post-test writing texts were independently scored by two experienced Chinese teachers familiar with GCSE writing standards. Both raters had more than five years of GCSE Chinese teaching experience. All essays were anonymized and randomly mixed during scoring. Inter-rater reliability was assessed using Pearson correlation coefficients: r = 0.975 (*p* < .001) for the pre-test and r = 0.962 (*p* < .001) for the post-test, indicating good reliability. Based on this, the average of the two raters’ scores was used as the student’s final writing score, as shown in Table 5.

## 5. Findings

The quantitative analyses compared the differences between the WL and no written languaging (NOWL) conditions on three writing accuracy indicators, and reported changes in overall writing scores from pre-test to post-test. These analyses address Research Questions 1 (immediate effects of WL on accuracy indicators) and 2 (transfer effects on overall writing performance). Qualitative analyses, based on WL texts and semi-structured interviews, examined learners’ foci of attention during feedback processing and their perceptions of the activities, addressing Research Question 3 (learner perceptions and subjective evaluations).

The presentation of findings is organized to reflect the hierarchical nature of the cognitive architecture hypothesized in the theoretical framework: we first report effects at the most local level (character accuracy), then at the text level (error density), then at the clause level (syntactic accuracy), followed by transfer effects (GCSE scores), and finally the qualitative evidence for cognitive processing mechanisms.

### 5.1. Quantitative Results

#### 5.1.1. Descriptive Statistics

Table 6 presents the descriptive statistics for the pre-test and post-test total scores, as well as the three writing accuracy indicators (character accuracy rate, errors per 100 characters, and proportion of error-free clauses) under the WL and NOWL conditions.

As shown in Table 6, participants’ GCSE writing total scores increased from 6.07 (SD = 2.22) at pre-test to 6.97 (SD = 2.18) at post-test, suggesting a positive trend in overall writing performance. For the accuracy indicators, the mean character accuracy rate under WL (M = 0.86, SD = 0.07) was higher than under NOWL (M = 0.82, SD = 0.08), indicating a modest advantage for WL at the orthographic level. The mean errors per 100 characters under WL (M = 0.11, SD = 0.05) was approximately half that under NOWL (M = 0.21, SD = 0.23), suggesting a more substantial reduction in overall error density when WL was employed. Notably, the standard deviation for this indicator under NOWL was notably larger (SD = 0.23 vs. 0.05), with a maximum of 1.00, indicating greater variability and the presence of extreme values among participants who did not engage in WL. This suggests that WL may have a stabilizing effect on writing performance, reducing the variability that characterizes unmediated feedback processing. The difference in the proportion of error-free clauses between the two conditions was small (WL: M = 0.27, SD = 0.16; NOWL: M = 0.24, SD = 0.15), suggesting that the WL effect may be less pronounced at the syntactic level.

#### 5.1.2. Normality Tests and Statistical Method Selection

To determine the appropriateness of parametric versus non-parametric tests, Shapiro–Wilk normality tests were conducted for all dependent variables. Given the small sample size (N = 15), this test provides a reasonably robust basis for normality judgement (Larson-Hall, 2016). Table 7 presents the normality test results.

The Shapiro–Wilk test showed that all variables except errors per 100 characters under the NOWL condition met the normality assumption (*p* > .05). Errors per 100 characters under NOWL significantly deviated from normality (W = 0.586, *p* < .001), consistent with the large standard deviation and extreme maximum value seen in the descriptive statistics. This violation is theoretically meaningful: it indicates that without WL, learners’ error density is not normally distributed some learners perform adequately while others produce a very high number of errors, reflecting the variability in cognitive processing depth that WL is designed to address. Therefore, this study selected the Wilcoxon signed-rank test as the primary inferential statistical method for all within-subject comparisons it does not require normality and is more robust in small-sample repeated-measures designs (Plonsky & Oswald, 2014).

#### 5.1.3. Immediate Effects of Written Languaging on Writing Accuracy

To address Research Question 1 whether WL produces immediate writing accuracy improvement beyond direct feedback Wilcoxon signed-rank tests were used to compare the three accuracy indicators between the WL and NOWL conditions. Results are presented in Table 8.

As shown in Table 7, the pattern of differences between the two conditions was not uniform across the three accuracy indicators. This differentiated pattern provides important evidence for the hierarchical nature of the cognitive architecture engaged by WL.

Character accuracy rate. The Wilcoxon test showed a trend approaching statistical significance between WL and NOWL (Z = −1.704, *p* = .088, r = 0.44), with a medium effect size. Rank distribution analysis showed that 11 out of 15 participants (73.3%) had higher character accuracy rates under WL than under NOWL, while only 4 showed the opposite pattern. This suggests a relatively consistent direction of change for most participants, although the effect did not reach conventional significance levels. The medium effect size (r = 0.44) indicates that the magnitude of the effect is practically meaningful, and the lack of statistical significance may be attributable to the small sample size rather than the absence of an effect. This near-significant trend suggests that WL may provide some benefit for orthographic processing—the most local level of cognitive control—but that a single WL session may not be sufficient to produce robust, statistically detectable improvement in character-level accuracy, which likely requires more extensive practice and consolidation (Shen, 2010; Ke, 1998).

Errors per 100 characters. There was a statistically significant difference between WL and NOWL (Z = −2.556, *p* = .011, r = 0.66), with a large effect size. This was the most robust finding among the three indicators. Rank distribution showed that 13 out of 15 participants (86.7%) had higher errors per 100 characters under NOWL than under WL, with only 2 showing the opposite pattern. The large discrepancy between the sums of positive and negative ranks (105.00 vs. 15.00) further supports the consistency and magnitude of this effect. This finding indicates that WL significantly reduces overall error density, suggesting that the metacognitive processing elicited by WL enhances learners’ ability to monitor and correct a broad range of errors across different linguistic levels. The large effect size (r = 0.66) underscores the practical significance of this finding.

Proportion of error-free clauses. No significant difference was found between the two conditions (Z = −0.534, *p* = .594, r = 0.14), with a small effect size. The rank distribution was relatively balanced: 8 participants performed better under WL, 6 performed better under NOWL, and 1 had equal scores. This balanced distribution suggests that WL did not produce a consistent effect on this indicator. This absence of effect at the clause level is theoretically significant: it suggests that the cognitive benefits of WL, while robust at the level of individual errors and overall error density, have not yet transferred to the more demanding level of producing completely error-free syntactic units. This hierarchical pattern significant effect at the text level, near-significant trend at the character level, and no effect at the clause level is consistent with the theoretical prediction that WL first strengthens local cognitive control (character and word-level processing) before extending to higher-level syntactic integration. The hierarchy observed here reflects a processing sequence that moves from lower-level, more automatic orthographic and lexical operations to higher-level, more controlled syntactic operations. Character-level processing demands fine-grained visuospatial analysis and retrieval of orthographic forms (J. Yang, 2022); vocabulary-level processing involves semantic selection and lexical collocation (Jiang, 2013); and clause-level processing requires the simultaneous coordination of orthography, lexis, word order, and grammatical relations—a more resource-intensive integration task (Sweller, 1988; DeKeyser, 2020). This hierarchical ordering is not merely an empirical observation but is theoretically grounded in the differential cognitive demands imposed by each linguistic level: lower levels require relatively discrete, self-contained operations, whereas higher levels require the integration of multiple lower-level outputs into a unified syntactic structure. This progression from local to global control is a well-documented pattern in skill acquisition more broadly (Anderson, 1983), and the differentiated WL effects observed here provide empirical support for this hierarchical processing model.

#### 5.1.4. Comparison of Overall Writing Scores from Pre-Test to Post-Test

To address Research Question 2 whether participants’ overall writing performance (as measured by GCSE total scores) changed significantly from pre-test to post-test Wilcoxon signed-rank tests were conducted on the pre-test and post-test total scores. Table 9 and Table 10 present the descriptive and inferential statistics, respectively.

As shown in Table 9 and Table 10, the post-test GCSE writing total score (M = 6.97, SD = 2.18) was higher than the pre-test (M = 6.07, SD = 2.22), with an average increase of 0.90 points. The Wilcoxon test showed a statistically significant difference between pre-test and post-test (Z = −3.342, *p* = .001, r = 0.89), with a large effect size. Importantly, 14 out of 15 participants (93.3%) had higher post-test scores than pre-test, none showed a decline, and one had the same score. This highly consistent direction of change indicates a positive effect of the instructional intervention on overall writing performance.

This transfer effect is particularly significant. The post-test was administered two weeks after the last WL activity, with no feedback provided during the post-test itself. The significant improvement suggests that the benefits of WL extended beyond the immediate task context. The cognitive processing elicited by WL appears to have strengthened knowledge representations and metacognitive strategies that remained accessible and effective even in the absence of immediate scaffolding. This is consistent with the theoretical prediction that WL promotes durable learning a hallmark of intelligent L2 acquisition rather than merely facilitating task-specific performance.

### 5.2. Qualitative Results

#### 5.2.1. Sources and Processing of Qualitative Data

Qualitative materials came from two sources: the record sheets completed by students during WL activities, and semi-structured interviews conducted with 5 participants after the experiment. The WL texts yielded 275 meaning units. The analysis used thematic content analysis (Schreier, 2012; Graneheim & Lundman, 2004), proceeding through three phases: segmentation into meaning units, initial coding, and category consolidation. The initial coding scheme referenced established categories from error analysis and second language writing research (Ferris, 2002) and was iteratively refined during analysis. The qualitative data provide crucial insight into the cognitive processes that underlie the quantitative findings specifically, how learners allocated their attentional resources during WL, and how individual differences in cognitive engagement moderated the effects of the intervention.

#### 5.2.2. Coding and Categorisation of Written Languaging Texts

Through constant comparative analysis (Glaser & Strauss, 1967), four core categories were induced from the 275 meaning units. Table 11 shows examples of initial labels, and Table 12 presents the frequency distribution of the four core categories.

The frequency distribution in Table 12 reveals a clear hierarchy in learners’ attentional allocation during WL: the majority of cognitive resources were devoted to character-level (48.4%) and word-level (29.8%) processing, with substantially less attention to syntactic issues (15.6%) and minimal attention to metalinguistic self-reflection (6.2%). This attentional hierarchy mirrors the hierarchical pattern of the quantitative findings and provides a cognitive explanation for why WL was most effective at reducing error density (which captures character and word-level errors) and least effective at improving clause-level accuracy (which requires syntactic coordination). Crucially, the qualitative data reveal that this hierarchy is not merely an artifact of measurement but reflects the actual allocation of learners’ limited cognitive resources during feedback processing. Learners spontaneously focused their languaging efforts on the linguistic levels that were most perceptually salient and most readily articulable character forms (visible, discrete) and word meanings (semantically accessible) while devoting comparatively little attention to syntactic structures, which require more abstract metalinguistic knowledge to articulate. This pattern is consistent with the predictions of cognitive load theory (Sweller, 1988): lower-level linguistic operations impose relatively low cognitive load and can be processed with minimal working memory resources, whereas higher-level syntactic integration demands substantial cognitive capacity, leaving fewer resources available for explicit metalinguistic reflection. The qualitative data thus provide direct evidence that the hierarchy observed in the quantitative outcomes corresponds to a hierarchy in learners’ attentional priorities and processing depth.

#### 5.2.3. Analysis of Written Languaging Texts

Formal attention at the character level. This was the most frequent category, accounting for nearly half of all coded units (133, 48.4%). Learners’ attention was predominantly focused on visible orthographic features: missing strokes, omitted radicals, reversed structure, and confusion of similar characters. For example, S8 pointed out: ‘The word ‘sea’ should be written as ‘each’, and I forgot the little dot next to it’ (误:每 → 正:海, with the missing dot on the right-hand component). These statements indicate that learners could go beyond simply being aware that they had written a character incorrectly, and could pinpoint exactly where the error occurred down to a specific stroke, dot, or local component. Another learner observed: ‘The stroke should have been on the left, but it was on the right’ (误:印 → 正:部, reflecting confusion in radical placement). Some of the responses also demonstrated an awareness of spatial arrangement: “The stroke should have been on the left, but it was on the right.” This indicates that the feedback not only prompted the learners to pay attention to individual components, but also to consider their relative positional relationships within the Chinese characters. This level of precision suggests that WL engages fine-grained visuospatial processing, which is a distinctive feature of logographic orthographic learning (J. Yang, 2022; X. Yan & Lin, 2023).

Semantic and usage attention at the word level. This was the second largest category, with 82 coded units (29.8%), involving attention to word meaning, appropriateness of word choice, and fixed expressions. Notably, some responses showed cross-linguistic awareness. S3 wrote: “High school refers to a middle school, not going to school. Because the meaning of ‘going to school’ is to attend school.” Another learner pointed out: “I wrote ‘white rice’, but it should be ‘rice’.” These examples suggest that learners were not simply replacing erroneous forms with correct ones, but actively considering semantic distinctions and usage conventions a form of hypothesis-testing that Swain and Lapkin (1995) identified as a key function of output. However, most word-level languaging remained at the level of correcting specific items, rather than rising to systematic rule generalisation, indicating that while WL prompts semantic processing, it does not always lead to the extraction of broader lexical principles.

Syntactic and structural attention. This category contained 43 coded units (15.6%). Although less frequent than the first two categories, languaging at this level often reflected deeper processing. Learners who engaged at this level attempted to reorganise sentence constituents or reflect on grammatical rules. For instance, one learner wrote: “It should be ‘I have many English classes’, rather than ‘I have a lot of English classes’.” Another learner pointed out: “In the sentence ‘I like China because China is very large’, ‘isn’t it’ is not needed.” These responses indicate that some learners could move beyond the word level to consider word order and grammatical functions. However, some responses reflected awareness of a problem but difficulty in articulating the specific issue: ‘The sentence combination is wrong, the two sentences are mixed together’ (e.g., 我喜欢中国因为中国很大. → 我喜欢中国因为中国是很大. where the copula 是 was incorrectly inserted).” This suggests that while learners perceived sentence-level problems, they did not always have sufficient metalinguistic resources to explain them fully a finding consistent with the limited syntactic attention observed in the frequency data.

Metalinguistic awareness and reflection on the learning process. The smallest category contained 17 coded units (6.2%), involving reflection on the learning process and task performance. These included statements such as “I wrote too fast, so I missed strokes” and “I haven’t reviewed the food unit for a long time.” One learner wrote: “I know what I want to say, but I haven’t learned how to write it in characters.” These responses reveal that learners sometimes connected their errors to their own cognitive states, writing speed, or prior learning experiences, rather than simply attributing errors to a lack of knowledge. This category is particularly significant from a cognitive architecture perspective, as it reflects emerging self-regulatory behavior—the ability to monitor one’s own learning processes and identify cognitive constraints. However, its low frequency (6.2%) suggests that such metalinguistic self-reflection is not automatically activated by WL but may require additional scaffolding or greater learner maturity.

#### 5.2.4. Interview Results

After the experiment, semi-structured interviews were conducted with 5 participants (S1–S5) to explore their perceptions of direct feedback and WL activities. Three themes emerged from the interviews, providing insight into how individual differences moderate the cognitive effects of WL.

Overall attitudes towards direct feedback and WL. All interviewees expressed positive attitudes towards direct feedback, finding it clear and helpful for identifying errors. S1 said: “The teacher’s feedback was timely; I could find errors I hadn’t noticed before.” S3: “Detailed corrections help me remember characters more easily.” However, attitudes towards WL were mixed. Some participants found it helpful for understanding errors, while others found it time-consuming and burdensome. S2 said: “I couldn’t even understand the feedback, so I couldn’t do the WL either.” This indicates that learners’ acceptance of WL may depend on their ability to understand the initial feedback a prerequisite cognitive condition that is not universally met.

Self-assessment of feedback understanding and processing. Participants varied considerably in their confidence in understanding feedback and making revisions. Some were able to identify feedback focus and revise independently: “After receiving feedback, I usually correct the wrong characters first” (S4). In contrast, others reported relying on surface strategies: “I just copy the character once, as long as it’s correct” (S2). This variation suggests that providing direct feedback alone does not guarantee deep processing for all learners. Those with stronger metalinguistic awareness engaged in more strategic, reflective processing, while those with weaker skills defaulted to shallow copying a pattern consistent with the individual difference literature in cognitive architecture (Skehan, 2015; Ishikawa & Suzuki, 2023).

Subjective evaluation of the pedagogical value of WL. Learners who engaged more deeply with the WL task tended to see its value. One participant (S5) said: “I write down what the teacher says, so I can be clearer about how to revise.” Another (S4) noted: “The teacher’s feedback points out my mistakes, and WL helps me remember. Using both together helps the most.” In contrast, participants with weaker Chinese proficiency or lower motivation viewed WL as an additional burden. S2 said: “It wasn’t helpful, it wasted time. I write slowly anyway, and having to write down what the teacher said was troublesome.” These contrasting views suggest that the effectiveness of WL is moderated by learners’ proficiency, self-efficacy, and motivation a finding that has important implications for the design of both human and AI-mediated feedback interventions.

In summary, the findings address the three research questions as follows:

RQ1 (Immediate effects): WL significantly reduced errors per 100 characters (*p* = .011, r = 0.66) with a large effect size, showed a near-significant positive trend for character accuracy (*p* = .088, r = 0.44), and had no significant effect on the proportion of error-free clauses (*p* = .594). This differentiated pattern reveals a hierarchical effect of WL, with benefits first manifesting at the level of local error correction and not yet transferring to higher-level syntactic accuracy.

RQ2 (Transfer effects): GCSE writing total scores improved significantly from pre-test to post-test (*p* = .001, r = 0.89), with 93.3% of participants showing improvement. This indicates that the benefits of WL transferred to subsequent writing performance, even in the absence of immediate feedback.

RQ3 (Learner perceptions): Learners’ attention during WL was predominantly focused on character-level (48.4%) and word-level (29.8%) issues, with limited syntactic attention (15.6%) and minimal metalinguistic self-reflection (6.2%). Interview data revealed that learner engagement with WL was moderated by proficiency and motivation, with higher-proficiency learners finding it beneficial and lower-proficiency learners perceiving it as burdensome.

These findings, viewed together, provide a coherent picture of the cognitive architecture of WL: it functions as a metacognitive amplifier that strengthens noticing and hypothesis-testing at local linguistic levels, producing measurable reductions in error density and transferable improvements in overall writing performance, but its effects are hierarchical and moderated by individual differences in cognitive and motivational resources.

## 6. Discussion

This study examined the effects of WL with direct feedback on writing accuracy among beginner CSL learners in a UK secondary school. The quantitative and qualitative findings jointly reveal a core finding: WL can effectively reduce writing error density, but its effect is clearly hierarchical, acting first on local linguistic features and not yet significantly improving overall syntactic error-freeness at the clause level. The following discussion addresses the three research questions in turn, integrating theoretical frameworks and existing literature to interpret this differentiated pattern, and concludes with implications for understanding the cognitive architecture of intelligent L2 learning and for the design of AI-assisted feedback systems.

### 6.1. Immediate Effects of Written Languaging on Writing Accuracy: A Hierarchical Cognitive Architecture

This finding provides empirical evidence for a core cognitive architecture: WL functions as a metacognitive amplifier that strengthens noticing and hypothesis-testing at local linguistic levels, producing measurable reductions in error density and transferable improvements in overall writing performance yet its effects are hierarchical and moderated by individual differences in cognitive and motivational resources. By ‘cognitive architecture,’ we refer specifically to the functional processing sequence—noticing → hypothesis-testing/metalinguistic reflection → consolidation that we operationalised through our WL task and measured through accuracy indicators. Our data are interpreted as behavioural manifestations of this architecture rather than direct observations of cognitive structures. The quantitative results showed that the errors per 100 characters under the WL condition were significantly lower than under the NOWL condition, with a large effect size (Z = −2.556, *p* = .011, r = 0.66), and 86.7% of participants showed a consistent direction of change. This finding aligns with previous studies indicating that WL promotes more successful revision of language errors (Suzuki, 2012; Moradian et al., 2017; Yılmaz, 2016).

First, activation of the output functions. From the perspective of the output hypothesis (Swain, 1985), the WL task required learners to articulate their reasoning about language forms, thereby activating the noticing/triggering, hypothesis-testing, and metalinguistic functions. When learners explained “why this form is incorrect,” they were compelled to attend to the gap between their own production and the target form a process that Swain and Lapkin (1995) considered critical for language development. The significant reduction in errors per 100 characters suggests that this languaging process did facilitate deeper cognitive processing of feedback information. Crucially, the WL texts themselves provide evidence of this activation: learners explicitly identified error types (“I forgot the dot”), proposed corrections (“it should be the character for ‘rise’“), and in some cases articulated the underlying rule (“in this sentence, ‘is’ does not need to be used”). This progression from noticing to hypothesis-testing to metalinguistic reflection from less to more cognitively demanding is precisely the sequence that the output hypothesis predicts.

Second, extension to a typologically distinct target language. Most previous research was conducted with adult learners in EFL settings where target language errors are predominantly morphosyntactic (e.g., tense, agreement). This study provides evidence that WL is also effective for adolescent beginners learning a typologically different language Chinese, with its logographic writing system and associated orthographic processing demands. The qualitative finding that 48.4% of WL units focused on character-level features indicates that WL effectively scaffolds the visuospatial analysis required for Chinese character learning. This extends the languaging construct beyond alphabetic contexts and suggests that WL can serve as a cognitive tool for orthographic as well as grammatical processing. Learners who wrote explanations such as “the character for ‘sea’ should be written as ‘each’, and I forgot the little dot” were engaging in a form of visual analysis that is specific to logographic writing systems—a cognitive process not previously examined in WL research.

Third, evidence of cross-task transfer. Unlike studies that only examined immediate revision on the same text (e.g., Suzuki, 2012), the crossover design used in this study involved new writing tasks, demonstrating that the benefits of WL are not limited to immediate revision but transfer to subsequent writing production. This transfer effect is critical for establishing WL as a strategy that promotes durable learning rather than merely task-specific performance. The significant improvement in GCSE scores from pre-test to post-test (Z = −3.342, *p* = .001, r = 0.89) provides particularly strong evidence for this transfer, as the post-test was administered two weeks after the last WL activity and with no feedback provided. This suggests that WL strengthened knowledge representations in a way that remained accessible and effective in a different performance context.

Fourth, the hierarchical pattern. The most theoretically significant finding is the differentiated pattern across the three accuracy indicators. The significant effect on errors per 100 characters (a global error density measure that captures errors at all levels) combined with the near-significant trend for character accuracy and the absence of effect for error-free clauses reveals a hierarchy in WL’s effects. This hierarchical pattern suggests that WL’s cognitive benefits first manifest at the level of reducing the frequency of local errors (character and word-level) before extending to the more demanding task of producing completely error-free syntactic units. This is consistent with the idea that L2 writing development proceeds from local to global control a process observed in other domains of skill acquisition (Sweller, 1988; DeKeyser, 2020). Character-level and word-level corrections are relatively discrete, self-contained cognitive operations that require attention to a single linguistic unit. In contrast, producing an error-free clause requires the simultaneous coordination of multiple linguistic dimensions orthography, lexis, syntax, and discourse within a single output unit. This is a higher-order integration task that may require more extensive practice and consolidation than a single WL session can provide.

Theoretical rationale for the hierarchical pattern. The hierarchical effect observed in this study significant reduction in errors per 100 characters, near-significant trend for character accuracy, and no effect on clause-level accuracy requires explicit theoretical grounding. We argue that this hierarchy reflects three distinct but interconnected dimensions of cognitive processing in L2 writing.

First, linguistic processing proceeds from local to global. Character-level processing is the most local: it involves the retrieval of orthographic forms and the execution of visuospatial configurations operations that are relatively discrete and self-contained (J. Yang, 2018; Chan et al., 2021). Vocabulary-level processing is intermediate: it involves semantic selection, collocational knowledge, and lexical constraints operations that require the integration of form and meaning but remain within the boundaries of individual lexical items (Jiang, 2013). Clause-level processing is the most global: it requires the simultaneous integration of orthographic, lexical, syntactic, and discourse information into a coherent syntactic unit (Polio & Shea, 2014). This progression from local to global processing is well-documented in both L2 acquisition research (Skehan, 2015) and cognitive skill acquisition theory (Anderson, 1983), which posits that skill development proceeds from declarative knowledge (knowing that) through proceduralisation to automaticity a process that typically begins with lower-level components before higher-level integration becomes fluent (DeKeyser, 2020).

Second, cognitive load increases with linguistic level. In cognitive load theory, the mental effort required to perform a task depends on the number of interacting elements that must be simultaneously processed in working memory (Sweller, 1988). Character-level processing involves a limited number of interacting features (radicals, strokes, spatial configuration); vocabulary-level processing involves a moderate number (semantic features, collocational constraints); but clause-level processing involves a large number of interacting elements (orthographic forms, lexical choices, word order, grammatical agreement, and discourse coherence). As a result, clause-level accuracy is the most cognitively demanding outcome to achieve, particularly for beginner learners whose working memory resources are already taxed by basic production demands (Muñoz, 2014). WL, as implemented in this study, reduced cognitive load at the local level by directing learners’ attention to discrete errors, but it did not, and could not, within a single brief intervention, provide the extensive practice needed to automatise the higher-level integration processes required for error-free clause production.

Third, the observed hierarchy reflects a developmental ordering. The three accuracy indicators are not merely different measures of the same construct; they represent different stages in the developmental progression of L2 writing accuracy (Jiang, 2013). Character-level accuracy is the foundational level: without reliable character recognition and production, higher-level syntactic processing is severely constrained. Vocabulary-level accuracy is the next layer: lexical choice and collocational knowledge mediate between orthographic forms and syntactic structures. Clause-level accuracy is the highest level: it presupposes reasonably stable control over the lower levels. This developmental ordering predicts that instructional interventions will first affect lower-level outcomes before higher-level outcomes show improvement, a prediction borne out by our data. The hierarchical pattern thus is not an arbitrary ordering but reflects a principled developmental sequence grounded in both linguistic theory and cognitive science.

Fourth, the qualitative data provide convergent evidence for this hierarchy. Learners’ WL texts showed that their spontaneous attentional focus followed the same hierarchy: character-level issues received the most attention (48.4%), vocabulary-level issues received moderate attention (29.8%), and syntactic issues received the least (15.6%). This attentional distribution is not merely a descriptive finding; it provides evidence that learners’ cognitive resources were allocated according to the same local-to-global hierarchy that we theorise. Learners attended most to the levels they could most readily identify and articulate, and least to the levels that required the most abstract metalinguistic knowledge.

In summary, the hierarchical effect is not an artefact or a premature interpretation. It is a theoretically grounded finding that reflects: the local-to-global organisation of linguistic processing, the differential cognitive load imposed by different linguistic levels, the developmental progression of L2 writing accuracy, and the attentional priorities evidenced in learners’ WL texts. The fact that WL’s effects were strongest at the text level (errors per 100 characters, which aggregates local errors), weaker at the character level, and absent at the clause level is precisely what a hierarchical cognitive architecture would predict.

This hierarchical interpretation is strongly supported by the qualitative data. Learners’ WL texts showed a clear attentional hierarchy: character-level issues (48.4%) received the most attention, followed by word-level issues (29.8%), with syntactic issues receiving substantially less (15.6%). This attentional distribution explains why error density which is heavily weighted toward character and word-level errors decreased significantly while clause-level accuracy which depends on syntactic integration did not improve correspondingly. The cognitive resources allocated by learners during WL were predominantly directed toward the local linguistic features that they could most readily identify and articulate. As learners gain more experience with WL, and as their proficiency develops, it is plausible that their attentional focus will gradually shift toward higher-level syntactic features a developmental trajectory that future longitudinal research should investigate. This finding carries a clear implication for AI design: an intelligent system should not be optimized solely for correction accuracy. Rather, it should be designed to elicit and capture such local-level cognitive engagement. For instance, by prompting learners to articulate *why* they made a specific character error, the system can move learners from passive receipt of correction to active orthographic analysis.

#### Hierarchical Effects and the Cognitive Architecture of L2 Feedback Processing

The hierarchical pattern observed in this study, local-level improvements (character accuracy, error density) without parallel gains in clause-level accuracy, raises a fundamental question: why does WL’s facilitative effect not transfer immediately to higher-level syntactic outcomes? We argue that the answer lies in the modular structure of the cognitive architecture that underlies L2 writing production.

L2 writing, viewed from a cognitive processing perspective, involves multiple processing modules that operate at different levels of linguistic representation (Levelt, 1989; DeKeyser, 2020). The orthographic module handles the retrieval and execution of character forms; the lexical module manages semantic selection and collocational constraints; and the syntactic module organises words into grammatically well-formed and discourse-appropriate structures. Crucially, these modules are hierarchically organised: lower-level modules must achieve a sufficient degree of automaticity before higher-level modules can function efficiently (Anderson, 1983; Segalowitz, 2010). This modular perspective predicts that instructional interventions that strengthen lower-level processing—such as WL’s facilitation of character-level noticing and correction, will improve lower-level outcomes relatively quickly, but higher-level syntactic integration will improve only after lower-level processing has become sufficiently automatised to free up working memory resources for syntactic planning and monitoring (Sweller, 1988).

The hierarchical pattern thus reflects a fundamental property of the cognitive architecture: the processing bottleneck at higher levels. Lower-level operations are relatively independent and can be improved through focused attention on discrete errors; higher-level operations require the coordination of multiple lower-level outputs, making them more resistant to short-term intervention. The fact that WL reduced errors per 100 characters, a measure that aggregates errors across all levels, while not affecting clause-level accuracy suggests that WL successfully strengthened local processing but that the strengthening was not yet sufficient to support consistent error-free syntactic production. This is consistent with the broader literature on L2 development, which shows that grammatical accuracy typically lags behind lexical and orthographic accuracy in the early stages of L2 writing development (Jiang, 2013).

Importantly, this hierarchical model has direct implications for the design of future interventions, including AI-mediated ones. If WL’s effects are inherently hierarchical, then instructional scaffolding should follow the same developmental sequence: first targeting character and word-level errors, then gradually shifting to syntactic structures as learners’ lower-level processing becomes more automatised. Adaptive AI systems could be designed to monitor learners’ performance across these different linguistic levels and to adjust the focus of languaging prompts accordingly.

### 6.2. Character Accuracy Rate: Interpreting the Positive Trend

The character accuracy rate approached but did not reach statistical significance (*p* = .088, r = 0.44). While 73.3% of participants showed improvement in character accuracy under the WL condition, the effect was not as robust as that for error density. Several theoretically relevant factors may explain this pattern.

First, the cumulative nature of character acquisition. Character acquisition is inherently cumulative, requiring repeated exposure and practice over time (Shen, 2010; Ke, 1998; J. Yang, 2018, 2022). Unlike grammatical rules that can be explained and applied relatively quickly, orthographic representations the precise visuospatial configurations of characters require multiple encounters with correct forms in varied contexts to become stable. A single instance of WL per task may not be sufficient to establish robust orthographic representations. The medium effect size (r = 0.44) suggests that the intervention did have a meaningful impact, but statistical power was limited by the small sample (N = 15). With a larger sample, this trend might reach conventional significance levels. Future research with larger samples and extended WL interventions would be needed to determine whether character accuracy is indeed amenable to WL or whether it requires different types of instructional support (e.g., explicit stroke-order training, radical analysis exercises).

Second, the nature of orthographic processing. The WL task required learners to articulate their reasoning about errors in language essentially, a verbal-linguistic activity. However, character learning involves a significant visuospatial component that may not be fully captured by verbal explanation (Chan et al., 2021). Learners might be able to identify errors and articulate what is wrong (e.g., “I wrote ‘big’ instead of ‘too’ because I forgot the dot”) but still lack the procedural, motor-based knowledge required to execute the correct character form consistently. This suggests that WL may be more effective for conscious, declarative aspects of orthographic knowledge (knowing *that* a character is wrong) than for procedural aspects (knowing *how* to produce it correctly). The qualitative data support this interpretation: several learners wrote statements such as “I don’t know what the Chinese characters for Beijing are” or “Although I know how to express it in a meaningful way, I haven’t learned how to write it,” revealing a gap between awareness and execution. For AI-assisted learning environments, this finding suggests that intelligent systems should not only prompt languaging about errors but also provide targeted practice on character formation through multimodal scaffolds (e.g., animated stroke-order demonstrations, radical decomposition exercises) that address the procedural dimension of orthographic learning.

Third, the role of practice and consolidation. The near-significant trend at the character level, combined with the significant effect at the error density level, suggests that WL may be more effective at *reducing the frequency* of character errors by helping learners notice and correct them than at eliminating them altogether. This is an important distinction. Error density is a global measure that can be improved by correcting some errors while leaving others uncorrected. Character accuracy, by contrast, requires that each individual character be produced correctly a more stringent standard. This pattern is consistent with the broader literature on skill acquisition, which shows that performance improvements typically emerge first in the reduction of errors (accuracy) before reaching complete error-free performance (fluency) (DeKeyser, 2020; Anderson, 1983). The findings suggest that WL is a valuable tool for the early stage of error reduction but may need to be supplemented with other interventions (e.g., repeated practice, distributed retrieval, targeted feedback on specific character types) to achieve complete elimination of character errors. For AI designers, this highlights the need for multimodal scaffolding. A system that simply provides a correct character may be insufficient. An intelligent system could integrate WL prompts with animated stroke-order demonstrations or radical decomposition exercises, thereby addressing the procedural dimension of learning that verbal languaging alone may not fully capture.

### 6.3. Proportion of Error-Free Clauses: Understanding the Absence of Effect

No significant effect was found for the proportion of error-free clauses (*p* = .594, r = 0.14), contrasting with the significant effect on the lower-level indicators. This absence of effect, while perhaps initially disappointing, is theoretically illuminating. It suggests that the benefits of WL are primarily confined to lower-level linguistic units (characters and individual words) and have not yet transferred to higher-level syntactic units. Understanding why this is the case provides crucial insights into the cognitive architecture of L2 writing and the role of WL within it.

First, cognitive load and resource allocation. Producing an error-free clause requires simultaneous control over multiple linguistic dimensions: orthography, lexical choice, word order, and grammatical structure. For beginner learners, each of these dimensions imposes significant cognitive load. WL, as implemented in this study, directed learners’ attention to individual errors but did not provide systematic training in syntactic coordination. The qualitative data show that only 15.6% of WL units involved syntactic and structural attention, and even when learners perceived sentence-level problems, they often struggled to articulate the specific issue (“The sentence combination is wrong, the two sentences are mixed together”). This suggests that the cognitive demands of clause-level accuracy exceed the processing resources that learners can allocate during a brief WL task. Learners may be able to identify a syntactic problem, but they lack the metalinguistic resources the explicit knowledge of grammatical rules and their interrelationships to fully articulate and resolve it.

Second, the hierarchical nature of language development. This finding aligns with previous research showing that the proportion of error-free clauses is a more stringent indicator and less sensitive to short-term interventions (Jiang, 2013). Jiang (2013) noted that judging error-free units in Chinese writing involves multiple dimensions syntax, vocabulary, and orthography making it a more demanding measure than simple error counts. The differentiated effect pattern in this study suggests that different accuracy indicators capture different aspects of language development at different stages. The benefits of WL may first manifest at lower levels (error reduction at the character and word levels) before gradually transferring to higher-level syntactic control—a process that may require more extended WL practice over multiple writing tasks and across different syntactic structures.

Third, implications for pedagogical sequencing. The hierarchical effect pattern has important pedagogical implications. It suggests that WL interventions should be sequenced developmentally: early WL tasks might focus on character and word-level errors—the types of errors that learners are most able to articulate and correct. As learners’ proficiency and metalinguistic awareness develop, WL tasks can gradually shift toward syntactic issues, prompting learners to explain not just individual corrections but the grammatical relationships among constituents. This developmental sequencing aligns with the broader principle of “scaffolding” (Wood et al., 1976) providing support that is matched to learners’ current level of competence and gradually withdrawing it as learners become more capable. For AI-assisted learning systems, this implies that feedback prompts should be adaptive: for beginner learners, prompts might focus on character and word-level errors; for more advanced learners, prompts could increasingly target syntactic structures and discourse-level coherence. Such adaptive scaffolding requires intelligent systems that can assess the learner’s current proficiency and adjust the cognitive demand of the languaging task accordingly.

Fourth, reconciling theoretical debates. The hierarchical pattern helps reconcile the long-standing debate between Truscott (1996) and Ferris (2004, 2010) regarding the value of corrective feedback. Truscott argued that grammar correction is ineffective because learners do not deeply process the feedback; Ferris countered that feedback can be effective when implemented appropriately. The present findings suggest that both perspectives capture part of the truth. Feedback without WL (NOWL) may indeed lead to shallow processing and limited learning, as Truscott feared. However, WL can deepen feedback processing and produce measurable improvements though these improvements are initially confined to lower-level linguistic features. The effects of feedback are not uniformly simultaneous across all levels but proceed in a hierarchical, stepwise manner. This hierarchical model of feedback processing provides a more nuanced theoretical account than either side of the debate alone.

### 6.4. From Immediate Revision to Cross-Task Transfer: Evidence for Durable Learning

The significant improvement in GCSE writing total scores from pre-test (M = 6.07) to post-test (M = 6.97) (Z = −3.342, *p* = .001, r = 0.89) indicates that the instructional intervention had a positive impact on overall writing performance, and this impact extended beyond the immediate task context. This finding is particularly compelling for three reasons.

First, the scope of transfer. The post-test used the same topic as the pre-test (“holiday experiences”) but was administered two weeks after the last WL activity, with no feedback provided during the post-test itself. The significant improvement suggests that learners had internalized some of the language forms and strategies that had been processed through WL, enabling them to produce more accurate writing even without immediate feedback. This is evidence not just of task-specific learning but of durable changes in the underlying cognitive representations that support writing performance. This aligns with the finding that WL promotes durable learning rather than merely task-specific performance (Suzuki, 2012; Yılmaz, 2016). The consistency of improvement (93.3% of participants showed higher post-test scores) further strengthens this interpretation.

Second, the nature of the GCSE assessment. The GCSE writing assessment criteria are holistic, covering both content and language quality. The significant improvement in GCSE scores indicates that the benefits of WL were sufficiently robust to be detected by a high-stakes, external assessment. This has important implications for practice: it suggests that WL is not merely a laboratory technique but a strategy that can improve performance on the authentic assessments that matter for learners’ educational outcomes. This finding should be of considerable interest to secondary school Chinese teachers seeking evidence-based strategies for improving GCSE writing performance.

Third, theoretical implications for cognitive architecture. The transfer effect suggests that WL contributed to strengthening the neural pathways and knowledge representations that underlie L2 production. By consolidating correct forms and their associated rules through written explanation, WL may have helped learners move from declarative knowledge (knowing that) toward procedural knowledge (knowing how) a shift that is central to the development of automaticity in L2 performance (Anderson, 1983; DeKeyser, 2020). The written record created during WL may also have served as an external memory aid that facilitated subsequent retrieval—a function that aligns with sociocultural theory’s emphasis on external mediation of cognitive processes (Lantolf & Thorne, 2006).

### 6.5. Learner Perceptions and Individual Differences: The Moderating Role of Cognitive and Affective Resources

The interview results revealed considerable individual variation in learners’ perceptions of WL, consistent with previous research on learner responses to feedback (S. Chen et al., 2016; Mahfoodh & Pandian, 2011). These variations provide important insights into the conditions under which WL is most effective and the mechanisms that mediate its effects.

Higher-proficiency learners appeared to benefit more from WL, whereas weaker learners struggled to engage meaningfully with the task. This pattern aligns with Fukuta et al.’s (2019) finding that the effects of WL may depend on learners’ proficiency. For learners who could understand the feedback and articulate their reasoning in writing (such as S5: “I write down what the teacher says, so I can be clearer about how to revise”), WL provided an opportunity for deep processing—a form of cognitive engagement that strengthened knowledge representations. In contrast, for learners who could not even understand the feedback itself (such as S2: “I couldn’t even understand the feedback, so I couldn’t do the WL either”), WL became an additional, unproductive burden rather than a supportive scaffold. This suggests that WL is most effective when learners possess the prerequisite linguistic and metalinguistic resources to benefit from it a finding consistent with the broader literature on aptitude-treatment interactions in L2 learning (Skehan, 2015; Ishikawa & Suzuki, 2023).

Motivation and engagement also moderated the effects of WL. Several participants expressed concerns about the time and effort required, with comments such as “It wasn’t helpful, it wasted time. I write slowly anyway” (S2). This highlights the importance of considering learners’ available cognitive and motivational resources when designing tasks. For learners with limited attention spans or low writing fluency, a full WL task may exceed their cognitive capacity, leading to superficial responses or disengagement. Conversely, learners who saw the value of WL (“Using both together helps the most”—S4) engaged more deeply and reaped greater benefits. This suggests that motivational interventions such as explaining the rationale for WL, reducing task demands for struggling learners, and providing positive feedback on languaging effort may be as important as the WL task itself.

This suggests that the effectiveness of WL is significantly moderated by learners’ cognitive and motivational resources. This finding is arguably the most critical for the development of personalized AI tutors. The ‘one-size-fits-all’ feedback approach, common in current AWE systems, is clearly suboptimal. An intelligent system must be adaptive. Based on the learner’s performance history and engagement patterns, it could: dynamically adjust the complexity of the languaging prompts; provide contingent feedback on the quality of the learner’s explanation; and offer differentiated support, such as sentence stems for struggling learners or more challenging metalinguistic questions for advanced learners.

### 6.6. Processing Mechanisms and the Nature of Intelligent L2 Learning

The integration of quantitative and qualitative findings provides a more complete picture of how WL affects writing accuracy and what it reveals about the cognitive architecture of intelligent L2 learning. Importantly, rather than re-stating the three theoretical functions of output, we ground our discussion in the empirical evidence from our qualitative data.

First, noticing amplification is evidenced by learners’ precise identification of orthographic features. Learners did not merely acknowledge errors but pinpointed specific visuospatial details, as exemplified by S8’s statement: “I wrote the character wrong, it should be the character for ‘rise’, not the one with only one mouth on the right”—and another learner’s observation: “the stroke should have been on the left, but it was on the right.” This level of fine-grained attention, extending beyond mere detection to explicit localization of missing radicals or misoriented strokes, demonstrates how WL amplifies and sustains the initial noticing triggered by direct feedback.

Second, hypothesis externalization and testing are observed when learners articulate structural rules and semantic distinctions. For instance, S3 wrote: “High school refers to a middle school, not going to school. Because the meaning of ‘going to school’ is to attend school.” Another learner noted: “It should be ‘I have many English classes’, rather than ‘I have a lot of English classes’.” These explanations go beyond simple replacement of erroneous forms; they reveal learners actively testing hypotheses about lexical collocations, semantic boundaries, and structural patterns, precisely the hypothesis-testing function that Swain and Lapkin (1995) theorized, now empirically traced in learners’ own words.

Third, metalinguistic reflection, though the least frequent category (6.2%), emerges when learners connect errors to their cognitive states or prior learning experiences. Examples include: “I wrote too fast, so I missed strokes,” and “I haven’t reviewed the food unit for a long time.” These statements indicate emerging self-regulatory behavior—the ability to monitor one’s own cognitive processes and identify the conditions under which errors occur. However, its low frequency suggests that such reflection does not occur automatically and may require additional scaffolding, particularly for adolescent learners whose metacognitive skills are still developing.

Fourth, the qualitative data also illuminate how individual differences moderate WL effects. Interview responses revealed that higher-proficiency learners found WL beneficial for understanding and remembering corrections (e.g., S5: “I write down what the teacher says, so I can be clearer about how to revise”), whereas lower-proficiency or less motivated learners perceived it as an additional burden (e.g., S2: “It wasn’t helpful, it wasted time”). This variation underscores that WL effectiveness is not uniform but depends on learners’ existing cognitive and motivational resources, a finding with direct implications for adaptive instructional design.

In summary, the evidence from this study indicates that WL, when combined with direct feedback, is an instructional strategy that can effectively promote deeper feedback processing among adolescent beginner CSL learners. Its effects are first evident in the reduction in identifiable local errors and gradually transfer to overall writing performance. However, the hierarchical nature of the effects also reminds us that the journey from “reducing errors” to “stable correctness” still requires longer practice and internalization processes. The cognitive architecture revealed by this study, a hierarchical system in which local error correction precedes higher-level syntactic integration, provides both empirical grounding and theoretical direction for future research and instructional design. Crucially, this cognitive architecture provides a blueprint for creating truly intelligent learning environments. The goal of AI, from this perspective, is not to replace the learner’s cognitive work, but to optimize its distribution—handling the “heavy lifting” of error detection while strategically offloading “meaning-making” back to the learner through adaptive WL prompts. The following section elaborates on the design principles that operationalize this vision.

### 6.7. Theoretical Implications for the Design of AI-Powered Feedback Systems

The hierarchical cognitive architecture revealed in this study carries potential implications for the design of intelligent writing systems. However, because our study did not implement or evaluate any AI tool, the following principles are theoretical extrapolations from our findings, not empirically validated design rules. They are offered to inform future research and development, not as tested prescriptions.

Current AWE and generative AI tools, while increasingly proficient at error detection, typically operate within a “detect-and-correct” paradigm. Our findings suggest that this paradigm may be cognitively suboptimal: it supplies the correct form but does not systematically engage the learner’s metacognitive architecture, thereby reinforcing the very “shallow processing” that WL is designed to mitigate (Truscott, 1996; Ferris, 2004). In contrast, the present findings support a “scaffold-and-elicit” paradigm, wherein AI systems could be engineered to optimize the distribution of cognitive labor between machine intelligence and human cognition. Specifically, the AI might handle the “heavy lifting” of pattern recognition and error detection, while strategically offloading the “meaning-making” work back to the learner through adaptive WL prompts. This paradigm translates into four empirically grounded design principles:

Hierarchical Prompting. The hierarchical pattern observed in our quantitative and qualitative findings, significant effects at the error density level, near-significant trends at the character level, and no effects at the clause level, provides empirical grounding for a developmental approach to WL prompting. This hierarchy reflects the differential cognitive demands of linguistic levels and the local-to-global organisation of L2 processing. The qualitative finding that 48.4% of learners’ attentional focus was on character-level features, and only 15.6% on syntax, indicates that WL prompts must be developmentally calibrated. For beginner learners, AI systems could initially target character and word-level errors (e.g., “What stroke or radical is missing?”), gradually escalating to syntactic prompts (e.g., “Can you explain the word order in this clause?”) as the learner’s proficiency and WL quality improve. One-size-fits-all prompts risk either overwhelming or under-challenging the learner.

Contingent Scaffolding via NLP. The near-significant trend for character accuracy (*p* = .088, r = 0.44) and the significant effect on error density (*p* = .011, r = 0.66) suggest that WL effectiveness depends on the *depth* of the explanation. An intelligent system equipped with NLP capabilities could assess the linguistic complexity and accuracy of the learner’s WL in real time (D. Yan & Zhang, 2024). If a learner produces a superficial explanation (e.g., “I got it wrong”), the system might issue a contingent follow-up prompt (e.g., “Can you identify *which* part of the character is incorrect and *why*?”). If the languaging is accurate and deep, the system could move to a more challenging syntactic prompt.

Adaptive Individualization. The interview data revealed that higher-proficiency learners found WL beneficial, while lower-proficiency learners perceived it as burdensome. This indicates that AI systems must be adaptive, differentiating prompt difficulty and task load based on the learner’s performance history and engagement patterns. For struggling learners, systems might provide sentence stems or reduced error foci (e.g., focusing only on two character errors), gradually increasing demands as self-efficacy and competence grow (Deci & Ryan, 2000). This personalization is beyond the reach of fixed-prompt designs.

Pedagogical Transparency. Learners’ mixed perceptions of WL (S2: “wasted time”; S5: “helps me remember”) suggest that AI systems could not merely require languaging but also provide metacognitive feedback on the *quality* of the languaging itself (e.g., “Your explanation is clear, this will help you remember this character”). This pedagogical transparency transforms the AI from a black-box corrector into a transparent cognitive partner.

These four principles, derived directly from the hierarchical effects and individual-difference patterns observed in this study, offer a theoretically grounded alternative to the current generation of AI writing tools. Future AI research should move beyond evaluating systems solely on correction accuracy and begin evaluating them on their capacity to foster sustained metacognitive engagement, a metric that aligns with the long-term goals of intelligent L2 development. In essence, the *intelligent language learner* is not defined by passive reception of correct forms, but by the active, self-regulated engagement with feedback, a capacity that our findings operationalize through the hierarchical architecture of WL. This architecture reveals that intelligence in L2 learning is not a fixed trait but a dynamic process of metacognitive regulation, which can be effectively scaffolded. For AI design, this implies a fundamental paradigm shift: from systems that *replace* cognitive labor (by providing answers) to systems that *redistribute* cognitive labor (by eliciting and amplifying the learner’s own reasoning). The four principles derived from our data, Hierarchical Prompting, Contingent Scaffolding, Adaptive Individualization, and Pedagogical Transparency, provide empirically grounded heuristics for building AI writing assistants that serve as genuine cognitive partners, capable of fostering the very metacognitive skills that underpin long-term, durable language development. Future experimental studies are needed to test whether such adaptive prompting actually enhances learning in AI-mediated environments.

## 7. Conclusions

This study contributes to the conceptualisation of the intelligent language learner by empirically investigating a core cognitive architecture—specifically, the noticing → languaging → internalisation processing sequence—operationalised through a WL task and measured through writing accuracy and transfer outcomes. While our behavioural data provide evidence for the operation of this architecture, we recognise that cognitive processes are inferred from, rather than directly observable in, these measures. We demonstrate that intelligence in L2 learning is not merely a trait or a fixed aptitude, but a capacity for self-regulated engagement with feedback a capacity that can be effectively scaffolded through WL. The resulting hierarchical model of feedback processing provides a theoretical contribution to SLA and offers empirically grounded insights that can inform the future development of adaptive AI-powered writing assistants. However, these implications are derived from a human-feedback study and should be tested in AI-mediated learning contexts before being translated into practice. By moving beyond the paradigm of error correction and embracing the paradigm of cognitive scaffolding, intelligent systems can be designed to cultivate the very metacognitive skills that underpin long-term, durable language development. In an era where AI tools are increasingly capable of providing correct answers, the distinctive value of human and human-centered AI learning lies precisely in the cultivation of learners’ capacity to reason about, reflect on, and self-regulate their own learning processes.

First, the effect of WL on writing accuracy is clearly hierarchical. Adding WL to direct feedback significantly reduced errors per 100 characters (Z = −2.556, *p* = .011, r = 0.66), with 86.7% of participants showing consistent improvement. However, the character accuracy rate showed only a positive trend without reaching significance (*p* = .088), and the proportion of error-free clauses showed no significant change (*p* = 0.594). This pattern indicates that the effect of WL is first manifested in the reduction of error density per unit of text, without simultaneously improving higher-level overall syntactic error-freeness. The qualitative data explain this hierarchy: learners’ attention during WL was predominantly focused on character-level (48.4%) and word-level (29.8%) issues, with limited syntactic attention (15.6%). This attentional distribution mirrors the quantitative hierarchy and provides a cognitive explanation for the differentiated effects.

Second, the benefits of WL transferred to subsequent writing performance. GCSE writing total scores improved significantly from pre-test to post-test (Z = −3.342, *p* = .001, r = 0.89), with 93.3% of participants scoring higher on the post-test. This suggests that some of the language knowledge processed through WL had been partially transformed from immediate revision into retrievable language resources, which continued to exert an inhibitory effect on errors when learners faced new writing tasks. This transfer effect is evidence that WL promotes durable, generalizable learning rather than mere task-specific performance.

Third, individual differences moderate the effects of WL. The interview results revealed that higher-proficiency learners tended to benefit more from WL, finding it helpful for understanding and remembering corrections. In contrast, lower-proficiency or less motivated learners viewed WL as an additional burden, struggling to engage meaningfully with the task. This suggests that the effectiveness of WL is not automatic but is significantly moderated by learners’ cognitive resources, proficiency, and motivation—a finding with important implications for both classroom practice and the design of AI-mediated learning environments.

Finally, WL functions as a metacognitive amplifier within the learner’s cognitive architecture. By deepening attention to errors, externalizing hypothesis-testing, and prompting metalinguistic reflection, WL enhances the cognitive processing of feedback beyond what is achieved by direct feedback alone. The hierarchical pattern of effects reveals that WL’s cognitive benefits first strengthen local, lower-level linguistic control before extending to higher-level syntactic integration. This hierarchical model provides a more nuanced understanding of how feedback processing unfolds in L2 writing and offers theoretical grounding for the development of adaptive, intelligence-informed instructional interventions.

This study has clearly identified the key components within the cognitive framework of intelligent second language learning, namely, the ability to transform feedback into learning through metacognitive language use. The findings demonstrate that “intelligence” in L2 learning is not merely about having high aptitude or using sophisticated strategies; it is fundamentally about the capacity for self-regulated, metacognitive engagement with linguistic input and feedback the very capacity that WL operationalizes. This study provides empirical evidence that such metacognitive capacity can be scaffolded through a simple, low-cost instructional intervention, with measurable effects on writing accuracy that transfer to subsequent performance. The study also identifies the boundaries of this scaffolding: WL is most effective at local levels of control and is moderated by individual differences, suggesting that future interventions whether human or AI-mediated must be adaptive to learners’ current cognitive states and developmental trajectories. Critically, the hierarchical nature of WL effects and the moderating role of individual differences identified in this study offer specific design parameters for AI systems. An intelligent writing assistant should not treat all learners identically; rather, it should assess learners’ proficiency and motivational states to determine the appropriate level of scaffolding; adapt WL prompts to target the linguistic levels (character, word, or syntax) that are most developmentally appropriate for the learner; and provide real-time, contingent feedback on the quality of learners’ languaging to deepen their metacognitive processing. These design principles, grounded in empirical evidence of the cognitive architecture of intelligent L2 learning, chart a path toward AI systems that are not merely intelligent in their outputs but genuinely intelligent in their capacity to foster human intelligence.

## 8. Limitations and Future Directions

This study has several limitations that suggest avenues for future research, particularly in the context of understanding intelligent L2 learning and the design of AI-assisted instructional systems.

First, AI integration and intelligent languaging scaffolds. Most importantly, this study did not involve any AI system; all feedback was provided by a human teacher. Therefore, our discussion of AI implications is speculative and theory-driven, not empirically grounded in AI-mediated interactions. We explicitly frame these discussions as future directions rather than as empirical contributions of the present work. This study was not an AI-intervention but provides the cognitive-psychological foundation for designing intelligent feedback systems. Future experimental studies could employ a three-arm comparison to empirically validate these principles: traditional AWE (corrections only), AWE + fixed WL prompts (standardized prompts across all learners), and AWE + adaptive WL (where prompt complexity and focus are dynamically adjusted to the learner’s proficiency, prior performance, and real-time languaging quality analyzed via NLP). This design would not only test the “scaffold-and-elicit” paradigm against the “detect-and-correct” paradigm but also provide crucial insights into the added value of adaptivity over fixed scaffolds. Recent advances in large language models (LLMs) make it increasingly feasible to generate contingent, contextually appropriate follow-up prompts, and to assess the depth of learners’ metalinguistic reasoning in real time (D. Yan & Zhang, 2024; Kohnke et al., 2023), enabling dynamic, personalized scaffolding that moves beyond one-size-fits-all feedback. Such research would directly translate the cognitive architecture identified in this study into an operational AI system, providing both theoretical insights into human–AI interaction and practical tools for the classroom. Furthermore, the absence of a no-intervention control group limits our ability to attribute pre-to-post improvements solely to the instructional intervention, as we cannot completely rule out the influence of maturation or practice effects over the study period. While the crossover design controls for individual differences and the two experimental conditions are equated for time on task, future research should include a no-feedback control group to more rigorously isolate the effects of WL from other temporal factors.

Second, sample size and generalizability. The small sample (N = 15) from a single UK secondary school limits statistical power and generalizability. The near-significant trend for character accuracy (*p* = .088, r = 0.44) may have reached significance with a larger sample. Future research should recruit larger, more diverse samples from multiple schools and L1 backgrounds to test the cross-context stability of the hierarchical effect pattern. Larger samples would also enable more sophisticated analyses of how individual differences (aptitude, working memory, motivation) moderate WL effects a crucial direction for understanding the cognitive architecture of intelligent L2 learning. Cross-linguistic comparisons (e.g., alphabetic vs. logographic target languages) would further illuminate whether the hierarchical pattern is specific to CSL or generalizes across writing systems.

Third, intervention duration and long-term effects. The short intervention (two WL sessions over two weeks) could not capture the cumulative developmental trajectory of WL effects, particularly at the syntactic level. The hierarchical pattern suggests that syntactic improvements may require extended practice across multiple writing tasks. Future longitudinal studies spanning a semester or academic year, with delayed post-tests at multiple intervals, are needed to assess whether WL’s effects consolidate over time and whether the observed hierarchy (local errors → global accuracy → syntactic control) represents a genuine developmental progression. Such research would also reveal the optimal frequency and intensity of WL interventions for durable learning.

Fourth, task authenticity and modality. The writing tasks were examination-oriented (GCSE materials), limiting generalizability to more open, communicative writing contexts. Additionally, the pen-and-paper WL format, while effective, may not be the most efficient or scalable modality. Future research should examine WL across diverse writing genres (narrative, expository, collaborative) and explore digital formats typed responses, voice-to-text languaging, or multimodal annotations. Digital WL offers advantages for automated collection, analysis, and adaptive scaffolding, and comparative studies of languaging modalities would inform both classroom practice and AI system design.

Fifth, learner engagement and individual differences. The finding that proficiency and motivation moderate WL effects highlight the need for research on enhancing engagement. Could brief training on WL strategies, gamification, or reducing cognitive demands (e.g., sentence stems, limiting error focus) increase participation, particularly for struggling learners? Research in educational psychology suggests that engagement can be enhanced through instructional design (Deci & Ryan, 2000) a principle applicable to WL. For AI systems, this implies adaptive prompting that matches task demands to learners’ current cognitive resources, ensuring that WL remains a scaffold rather than a burden.

Sixth, process-tracing and neurocognitive methods. The qualitative data (WL texts and interviews) provide indirect evidence of cognitive processes. Future research should employ think-aloud protocols, eye-tracking, or EEG to capture real-time cognitive activity during WL. Such process-tracing data would provide a more direct window into the cognitive architecture of WL how attention is allocated, how hypothesis-testing unfolds, and how metalinguistic reflection emerges over time. This would not only strengthen theoretical understanding but also inform the design of AI systems that can detect and respond to learners’ cognitive states in real time.

In summary, while this study provides robust evidence for WL as a metacognitive tool that enhances feedback processing in beginner CSL writing, these limitations point to clear directions for future research. Addressing them through larger samples, longitudinal designs, diverse tasks and modalities, AI integration, engagement interventions, and process-tracing methods will deepen our understanding of the cognitive architecture of intelligent L2 learning and enable the development of more effective, adaptive instructional systems.

## Figures and Tables

**Table 1:** Arrangement of two writing tasks and treatment conditions.

Sequence Group	Round 1 Task	Round 2 Task
Group 1 (*n* = 7)	Condition A: Direct feedback + WL	Condition B: Direct feedback only
Group 2 (*n* = 8)	Condition B: Direct feedback only	Condition A: Direct feedback + WL

Note: Condition A = WL; Condition B = NOWL.

**Table 2:** Study design overview.

Week	Activity	Sequence Group 1 (*n* = 7)	Sequence Group 2 (*n* = 8)	Purpose
Week 1	Orientation & Consent	All participants	All participants	Informed consent, proficiency confirmation
Week 2	Pre-test	Holiday topic	Holiday topic	Baseline writing score & accuracy (no feedback)
Week 3	Experimental Task 1	China travel topic + Condition A (WL)	China travel topic + Condition B (NOWL)	Immediate effect under first condition
Week 4	Experimental Task 2	School life topic + Condition B (NOWL)	School life topic + Condition A (WL)	Immediate effect under second condition (crossed)
Week 5	Post-test	Holiday topic	Holiday topic	Transfer effect (no feedback)
Week 6	Interviews	5 volunteers	5 volunteers	Qualitative perceptions

**Table 3:** Basic information of research participants.

ID	Gender	Age	L1	Years of Chinese Study	Current Course Level
S1	M	15	English	2	GCSE Mandarin (Foundation)
S2	F	15	English	2	GCSE Mandarin (Foundation)
S3	F	15	English	2	GCSE Mandarin (Foundation)
S4	M	15	English	2	GCSE Mandarin (Foundation)
S5	F	15	English	2	GCSE Mandarin (Foundation)
S6	F	14	English	2	GCSE Mandarin (Foundation)
S7	M	15	English	2	GCSE Mandarin (Foundation)
S8	F	15	English	2	GCSE Mandarin (Foundation)
S9	F	15	English	2	GCSE Mandarin (Foundation)
S10	F	15	English	2	GCSE Mandarin (Foundation)
S11	M	15	English	2	GCSE Mandarin (Foundation)
S12	M	15	English	2	GCSE Mandarin (Foundation)
S13	M	15	English	2	GCSE Mandarin (Foundation)
S14	F	15	English	2	GCSE Mandarin (Foundation)
S15	F	15	English	2	GCSE Mandarin (Foundation)

Note: S indicates student ID; course level is based on the school’s GCSE programme.

**Table 4:** Information on interview participants.

ID	Gender	Age	L1	Years of Chinese Study
S1	M	15	English	2
S2	F	15	English	2
S3	F	15	English	2
S4	M	15	English	2
S5	F	15	English	2

**Table 5:** Inter-rater reliability test results for pre-test and post-test writing total scores.

Test Phase	Variable	Correlation Type	Correlation (r)	Significance (2-Tailed *p*)
Pre-test	Writing total score	Pearson	0.975 **	<.001
Post-test	Writing total score	Pearson	0.962 **	<.001

Note: ** *p* < .01.

**Table 6:** Descriptive statistics for main variables.

Dimension	Variable	Condition/Phase	M	SD	Min	Max
Overall performance	Writing total score	Pre-test	6.07	2.22	2.00	9.00
	GCSE	Post-test	6.97	2.18	3.00	10.00
Character performance	Character accuracy rate	WL	0.86	0.07	0.69	0.96
		NOWL	0.82	0.08	0.65	0.95
Error density	Errors per 100 characters	WL	0.11	0.05	0.00	0.21
		NOWL	0.21	0.23	0.05	1.00
Linguistic accuracy	Proportion of error-free clauses	WL	0.27	0.16	0.00	0.60
		NOWL	0.24	0.15	0.00	0.47

**Table 7:** Normality test results for main variables.

Dimension	Variable	Shapiro–Wilk Statistic	Significance (*p*)	Normality Judgement
Overall score	Pre-test mean score	0.930	.273	Yes
	Post-test mean score	0.932	.292	Yes
WL condition	Character accuracy rate	0.940	.380	Yes
	Errors per 100 characters	0.943	.416	Yes
	Error-free clause proportion	0.969	.847	Yes
NOWL condition	Character accuracy rate	0.923	.214	Yes
	Errors per 100 characters	0.586	.000	No
	Error-free clause proportion	0.942	.408	Yes

Note: *p* > .05 indicates normality assumption met.

**Table 8:** Wilcoxon signed-rank test results for writing accuracy indicators across conditions.

Indicator	Ranks	N	Mean Rank	Sum of Ranks	Z	Sig. (*p*)	Effect Size (r)
Errors per 100 characters	Positive ranks (NOWL > WL)	13	8.08	105.00	−2.556	.011 *	0.66
	Negative ranks (NOWL < WL)	2	7.50	15.00			
Character accuracy rate	Positive ranks (WL < NOWL)	4	7.50	30.00			0.44
	Negative ranks (WL > NOWL)	11	8.18	90.00	−1.704	.088	
Error-free clause proportion	Positive ranks (WL < NOWL)	6	7.33	44.00			0.14
	Negative ranks (WL > NOWL)	8	7.63	61.00	−0.534	.594	

Note: Direction of comparison is based on SPSS output; Ties (equal scores) were excluded in calculating Z and are not repeated in the table; Effect size formula: r = |Z|/√N, * *p* < .05.

**Table 9:** Descriptive statistics for pre-test and post-test.

Dimension	Variable	Condition/Phase	Mean (M)	SD	Min	Max
Overall performance	Writing total score	Pre-test	6.07	2.22	2.00	9.00
	(GCSE)	Post-test	6.97	2.18	3.00	10.00

**Table 10:** Wilcoxon signed-rank test results for pre-test and post-test writing total scores.

Indicator	Ranks	N	Mean Rank	Sum of Ranks	Z	Sig. (2-Tailed)	Effect Size (r)
Writing total score	Positive ranks (post > pre)	14	7.50	105.00	−3.342	.001 **	0.89
(post-final-pre-final)	Negative ranks (post < pre)	0	0.00	0.00			
	Ties (post = pre)	1					

Note: Z is based on negative ranks; ** *p* < .01; Effect size formula: $r\;=\;|Z|/\sqrt{N}$.

**Table 11:** Examples of initial labelling results.

Original Text (Excerpts)	Segmented Meaning Unit	Initial Label
I wrote the character wrong, it should be the character for “rise”, not the one with only one mouth on the right.	Wrote the character wrong	Awareness of character writing errors
	It should be the character for “rise”	Recognition of correct character
	Not the one with only one mouth on the right	Identification of missing radical
I wrote the words in reverse order, the correct order should be “friends”, not the reversed sequence.	I wrote the words in reverse	Reversed order of words
I like China because it is very large. In this sentence, “is” does not need to be used.	In this sentence	Identification of sentence expression issues
	Don’t use is	Identification of copula misuse
I didn’t have much time to finish the content and sentences.	Didn’t have time	Time pressure
	Finish the content and sentences	Task completion difficulty
The weather is extremely hot. I made a mistake and wrote a similar-looking character instead.	The writing was incorrect.	Confusion of similar characters/radicals
I don’t know what the Chinese characters are in Beijing.	Not knowing the Chinese characters in Beijing	Recognition of proper noun writing difficulty
When writing about eating too much, it often becomes overly exaggerated, and one forgets to consider the actual situation.	Wrote “big” instead of “too”	Recognition of missing Chinese character strokes
	Forgot the dot	Insufficient monitoring of subtle features
I don’t know which Chinese character “in” is.	I don’t know which Chinese character “in” is	Identification of semantic correspondence obstacles in prepositions

**Table 12:** Frequency of core categories.

Core Category	Definition	Included Content	Count	Percentage
Formal attention at character level	Attention to character structure, strokes, radicals, and orthographic configuration	Wrong character, missing stroke, missing radical, structural errors, reversed direction	133	48.4%
Semantic and usage attention at word level	Attention to word meaning, word choice, word order, and naturalness of expression	Semantic errors, incorrect lexical construction, word order reversal, unnatural expression	82	29.8%
Syntactic and grammatical attention	Attention to word order, clause structure, and grammatical rules	Word order errors, sentence structure problems, classifier misuse, negation errors, “to be” misuse	43	15.6%
Metalinguistic awareness and reflection on learning process	Reflection on one’s own understanding process, expression difficulties, and task completion	Don’t know how to write, forgot, time pressure, task completion difficulty, self-improvement awareness	17	6.2%

Note: Counts and percentages are calculated based on segmented meaning units.

## Data Availability

Data will be made available on request. Authors are willing to share any data that are used in this work. All the data that support the findings of the study are provided.
